# Genome-wide association studies for identification of stripe rust resistance loci in diverse wheat genotypes

**DOI:** 10.3389/fpls.2025.1687331

**Published:** 2025-12-09

**Authors:** Vikesh Tanwar, Satish Kumar, Chuni Lal, Rajesh Aggarwal, Rajitha Nair, Disha Kamboj, Prem Lal Kashyap, Vikram Singh, Johar Singh Saini, Sunil Kashyap, Shabir Hussain Wani, Sripada M. Udupa, Rajender Singh, Ratan Tiwari

**Affiliations:** 1 Indian Council of Agricultural Research (ICAR)-Indian Institute of Wheat and Barley Research, Karnal, India; 2 Chaudhary Charan Singh (CCS) Haryana Agricultural University, Hisar, India; 3 Punjab Agricultural University, Regional Research Station, Gurdaspur, India; 4 Sher-e-Kashmir University of Agricultural Sciences and Technology-Kashmir (SKUAST-K), Srinaga, India; 5 International Centre for Agricultural Research in the Dry Areas, Rabat, Morocco

**Keywords:** wheat, biotic stress, stripe rust, screening, resistance

## Abstract

**Introduction:**

In North India, *Puccinia striiformis* f. sp. *triticii* (Pst), the causal agent of stripe rust, poses a significant challenge to wheat productivity. The frequent emergence of new virulent *Pst* strains has rendered many resistance genes ineffective. Hence, continuous identification and deployment of novel resistance genes are crucial for maintaining durable resistance and ensuring sustainable wheat cultivation.

**Materials and Methods:**

A genome-wide association study (GWAS) was conducted on 652 elite, diverse wheat genotypes using 1,938 DArTseq SNP markers. Field evaluations were performed at the adult plant stage across four locations—Hisar, Karnal, Gurdaspur, and Khudwani—under natural disease conditions. Marker–trait associations were identified using General Linear Model (GLM), Mixed Linear Model (MLM), and FarmCPU approaches, considering loci with –log₁₀(p) ≥ 3 as significant.

**Results:**

Analysis revealed 27 genomic regions significantly associated with stripe rust resistance across environments. Among these, four loci were located on chromosomes 2B and 6B, and three on 6A. Several loci corresponded to resistance-related genes, including NBS-LRR, F-box, LRR, protein kinase, Ser/Thr_kinase, Znf_RING-CH, E3-ubiquitin ligase, and ABC transporter genes, suggesting their potential role in rust resistance mechanisms.

**Discussion:**

The study identified novel genomic regions associated with Pst resistance, providing valuable resources for wheat improvement. The functional annotation of these loci highlights their involvement in plant defense pathways. Conversion of these loci into breeder-friendly molecular markers will facilitate marker-assisted selection (MAS) and accelerate the development of durable stripe rust-resistant wheat cultivars suited to North Indian agro-ecological conditions.

## Introduction

Wheat (*Triticum aestivum* L.) is a key staple crop that plays an essential role in feeding the global population and supporting food security. It is cultivated worldwide over an area of approximately 220 million hectares, yielding approximately 775.4 million tonnes of annual production (Prospects, 2022). By 2050, the global population is expected to reach approximately 9 billion, leading to a significant increase in demand for wheat. This crop is a vital source of daily caloric and protein intake, accounting for over 20% of caloric consumption and 25% of the protein on a global scale (Hubert et al., 2010). Nevertheless, wheat cultivation faces significant abiotic and biotic challenges that result in considerable reductions in yield and quality (Trethowan et al., 2018). According to Savary et al. (2019), it is estimated that diseases alone could lead to yield losses of approximately 21.5%. Among major wheat diseases, rusts are the most prevalent and damaging threats to wheat crops, affecting all regions where wheat is cultivated.

Among the various types of rust, stripe rust, commonly known as yellow rust, is a major threat to bread wheat (*T. aestivum* L.) on a global scale, caused by the fungus *Puccinia striiformis* f. sp. *tritici* (*Pst*). This disease significantly impacts wheat production worldwide. An outbreak of this disease rapidly devastates green leaves, leading to a drastic reduction in photosynthesis. Consequently, plants become weakened and stunted, resulting in fewer grains per spike, shriveled grains, and lower grain weights. According to Chen (2020), fields planted with susceptible cultivars are at high risk, with potential grain yield losses reaching up to 100%. Currently, 5.47 million tons of annual global losses in grain yield occur due to this disease (Beddow et al., 2015). Some fungicides effectively control this disease, but their use typically leads to higher crop production costs and poses significant environmental risks. Therefore, harnessing genetic resistance is the most powerful, cost-effective, and environmentally sustainable strategy for effectively combating this disease (Mapuranga et al., 2022). It is essential for researchers to actively seek out new resistance sources and incorporate innovative resistance genes into cultivars to stay ahead of emerging races of stripe rust. This proactive strategy will not only control the disease but also eliminate the “boom and bust cycle” associated with the performance of cultivars.

According to Van der Plank (Parlevliet and Zadoks, 1977), plant resistance is classified into two primary types—horizontal and vertical—which are determined by the interaction between the pathogen and host. Horizontal resistance, often referred to as partial resistance or non-specific resistance, is characterized by its ability to provide intermediate protection against a broad range of pathogens. This type of resistance is regulated by multiple genes and is fundamentally quantitative, resulting in various levels of resistance rather than absolute immunity. Furthermore, several biochemical and physiological processes, including the production of antimicrobial compounds and the reinforcement of cell walls, significantly contribute to the effective inhibition of pathogen colonization (Dyakov, 2007). Vertical resistance, also referred to as complete or specific resistance, represents a defense mechanism inherent in plants, providing robust protection against specific strains of pathogens or a limited range of closely related strains. This form of resistance is primarily regulated by one or a few genes, offering the plant comprehensive immunity to the particular pathogen in question. Specific resistance (R) genes in the plant’s genome enable the synthesis of proteins that recognize and interact directly with pathogen compounds, resulting in a prompt and accurate defensive reaction. This defensive response may encompass the activation of defense-related genes, the synthesis of toxic compounds, or the induction of localized cell death at the infection site, all of which serve to inhibit the pathogen’s proliferation (Orton, 2020). Chen (2005) classified host resistance into two categories: adult-plant resistance (APR), which is defined as horizontal-type resistance and regulated by numerous genes, and seedling or all-stage resistance (ASR), which is controlled by one or a few genes, similar to vertical-type resistance. ASR is effective during all growth stages and is typically characterized as having qualitative or monogenic resistance. ASR adheres to the gene-for-gene model described by Flor (1971), offering high levels of protection, but it is race-specific, and its effectiveness is compromised by high selection pressure on the pathogen, which may mutate to overcome resistance. In contrast, APR is more durable but often provides partial resistance. It is expressed or enhanced at the adult plant stage (Lagudah, 2011; Chen, 2013; Mundt, 2014; Ellis et al., 2014). Moreover, APR is usually non-race specific, although race-specific APR has also been identified (Milus et al., 2015). Despite their durability, APR genes do not protect plants at the seedling stage and tend to show variability in the timing and levels of resistance across environments, where a single APR gene often provides insufficient protection under severe epidemics (Risk et al., 2012; Chen, 2014; Singh et al., 2015). Pyramiding multiple APR genes is essential to provide a high level of resistance through additive or epistatic effects (Sørensen et al., 2014). Plant breeders seek to incorporate both forms of resistance into cultivars through systematic breeding programs to improve disease resistance in crops. In the agricultural sector, this approach is fundamental to integrated and sustainable pest management techniques (Sharma et al., 2025).

Eighty-seven genes are resistant to stripe rust (*Yr1* to *Yr87*), and over 350 quantitative trait loci (QTLs) have been identified and mapped to the wheat genome thus far (McIntosh et al., 2020; Zhu et al., 2023; Sharma et al., 2024). Out of the total named stripe rust resistance genes, 59 provide resistance at the seedling stage, while 28 provide resistance at the adult plant stage (APR). To date, only 12 *Yr* genes have been cloned, namely, *Yr5*, *Yr7*, *Yr10* (*YrNAM*), *Yr15*, *Yr18*, *Yr27*, *Yr36*, *Yr46*, *YrSP*, *YrAS2388*, *YrU1*, and *Yr87* (Fu et al., 2009; Krattinger et al., 2009; Liu et al., 2014; Moore et al., 2015; Marchal et al., 2018; Klymiuk et al., 2018; Zhang et al., 2019; Wang et al., 2020; Athiyannan et al., 2022; Ni et al., 2023; Sharma et al., 2024), of which *Yr18*, *Yr36*, and *Yr46* are APR genes. Generally, ASR genes are associated with nucleotide-binding domain and leucine-rich repeat proteins (Sánchez-Martín and Keller, 2021). These proteins recognize effector proteins produced by the pathogen to initiate effector-triggered immunity, thereby protecting the host (Gururani et al., 2012). In contrast, APR genes lack specific structural domains, feature more complex structures, and indirectly contribute to resistance (Sánchez-Martín and Keller, 2021). For example, *Yr18* encodes an ATP-binding cassette (ABC) transporter, *Yr36* encodes a protein kinase (WKS1), and *Yr46* encodes a hexose transporter. Most ASR genes and some APR genes deployed in commercial wheat cultivars are no longer effective due to the emergence of virulent *Pst* races (Hovmøller et al., 2011; Sørensen et al., 2014; Wan and Chen, 2014). Therefore, finding user-friendly markers becomes even more necessary (Zeng et al., 2019).

With the sequencing technology advancements in recent years, this has facilitated the development of innovative genotyping approaches that provide cost-effective and high-throughput capabilities for marker systems. Among these methods, Diversity Array Technology Single Nucleotide Polymorphism (DArTSNP) markers are particularly well-suited for diversity analysis, marker-assisted genetic resource management, marker-assisted selection, and genome-wide association studies (GWASs) in breeding programs. Through GWASs, progress in the identification of genes associated with stripe rust has been made in recent years. This is a robust methodology for elucidating the genetic foundation of complex traits. By analyzing extensive genetic data from diverse populations, researchers have identified specific genomic regions associated with resistance to stripe rust. These studies led to the discovery of novel genes and alleles of resistance that contribute to various agronomic traits, including drought and heat tolerance (Tadesse et al., 2019; Ballesta et al., 2019; Gudi et al., 2024, Gudi et al., 2025) as well as disease resistance (Kankwatsa et al., 2017; Kang et al., 2020) and grain quality characteristics (Tadesse et al., 2015). Many other complex agronomic traits in bread wheat provide valuable insights into understanding the plant immunity mechanisms. Finding these genes is a crucial step in creating improved and better cultivars with strong resistance to stripe rust, which will result in far more productive and sustainable agricultural practices. According to Brachi et al. (2011), GWASs effectively overcome the common challenges associated with bi-parental QTL mapping, such as restricted allelic diversity and limited genomic regions. The genetic architecture of complex traits in diverse germplasm collections can be studied using GWASs, which detect the genomic regions present in linkage disequilibrium (LD) with genes associated with the trait under study (Hall et al., 2010; Zhao et al., 2011; Riedelsheimer et al., 2012; Singh et al., 2025). Also, GWASs have been utilized to detect stripe rust resistance loci in different market classes of wheat (Naruoka et al., 2015; Liu et al., 2018; Liu et al., 2020; Mu et al., 2020; Muleta et al., 2020; Aoun et al., 2021; Zhang et al., 2021; Jambuthenne et al., 2022; El-Messoadi et al., 2024; Gao et al., 2024; Qiao et al., 2024). This study, consisting of 652 genotypes, also focused on identifying diverse wheat genotypes that revealed strong resistance at the adult plant stage to stripe rust, as well as finding genomic regions associated with stripe rust resistance through GWASs. A large and diverse set of wheat germplasm, including pre-bred materials, registered genetic stocks, and landraces, were used to dissect the variation for stripe rust resistance across four hotspot locations. A mid-density Single Nucleotide Polymorphism (SNP) marker panel was also used to scan the entire genome, which will reveal potentially novel QTLs that are different from known resistance genes. These identified QTLs can be further used for marker-assisted selection to develop improved, resistant wheat cultivars.

## Materials and methods

### Plant materials

The present study comprised a diverse set of 652 wheat genotypes. These genotypes included varieties (389), advanced breeding lines (53), genetic stocks (80), landraces (103), exotic lines (1), and mutants (24) (**Supplementary Table 1**). Genotypes in the panel are adapted to different agro-climatic zones of India, and this panel includes genotypes from all over the world. The stripe rust disease was evaluated in the field at four locations, namely, Karnal, Hisar, Gurdaspur, and Khudwani, in India during 2022–2023 (**Table 1**). These locations are in the stripe rust-prone areas of Northern India, which are suitable for studying natural disease pressure. These genotypes were obtained from the Germplasm Resource Unit (GRU), ICAR-IIWBR, Karnal, India.

**Table 1:** Field and geographical location of all the locations where experiment was performed.

Field location	Geographical location
New Farm, ICAR-IIWBR, Karnal, India	29°42′10.0″N, 76°59 29.7″E
Wheat and Barley Section, CCSHAU, Hisar, India	29°8 22.1928″ N, 75°4253.8812 E
RRS, PAU Gurdaspur, India	30°03′N, 75°27′E
RRS, SKAUST, Khudwani, India	34°17′N, 75°27′E

### Stripe rust evaluation at the adult plant stage in the field

The genotypes were sown in an augmented block design with 1-m length of a single row and 0.4-m distance between the rows. Sowing was performed in the first fortnight of November 2022–2023 at all four locations mentioned above. A variety of check lines that are known to be vulnerable to different rust infections were planted across the plot in infector rows (every 20th single row) and spreader rows (perpendicular to the 1-m rows). This approach establishes a robust inoculum, ensuring uniform disease development throughout the area. To achieve optimal disease distribution, collection was performed to capture naturally occurring disease during early infection in the spreader rows and used to inoculate the infector rows. The response to rust disease infection was measured using two parameters, namely, disease severity (DS) and infection response (IR). DS was assessed using the modified Cobb’s scale (Peterson et al., 1948), which estimated the percentage coverage of rust pustules (uredinia) on the flag leaf. The scale ranged from 0 to 100, with 0 indicating no coverage and 100 indicating full coverage. IR was evaluated by measuring the host’s reaction to rust pustules and converting it into a 0–1 scale (Roelfs et al., 1992). A fifth group, not mentioned by Roelfs et al. (1992), was classified for lines with a mixed response, ranging from moderately resistant to moderately susceptible. Rust response was evaluated using five scoring categories: resistant (R) at 0.2, moderately resistant (MR) at 0.4, mixed response (M) at 0.6, moderately susceptible (MS) at 0.8, and susceptible (S) at 1. Data were collected weekly, and three observations were made when the disease score of 60S (DS: 60; IR: S) was reached by the flag leaves of susceptible checks. Out of these multiple scores of a test line, the one with the score tending toward susceptibility from the last week was kept for the study. These scores were then utilized for GWASs across each environment by consolidating the two metrics into a single value known as the coefficient of infection (CI). This value was calculated as the product of DS and infection rate (IR) on a linear scale of 0 to 100, following the established methodologies of Loegering (1959) and Roelfs et al. (1992). Genotypes with CI scores of 0 to 1 were considered immune, 1–5 highly resistant, 5–15 moderately resistant, 15–25 moderately susceptible, and >25 susceptible. As a key trait for detecting significant marker–trait associations (MTAs), CI is very well-suited for GWASs and efficiently integrates data from both DS and IR about rust response (Yu et al., 2012; Gao et al., 2017; Mihalyov et al., 2017).

### Statistical analysis

#### ANOVA

An augmented RCBD package was used to analyze variance [analysis of variance (ANOVA)] for 652 genotypes across all locations using the R software (*https://www.r-project.org*; Aravind et al., 2020).

### DNA isolation and genotyping

**Figure 1:**
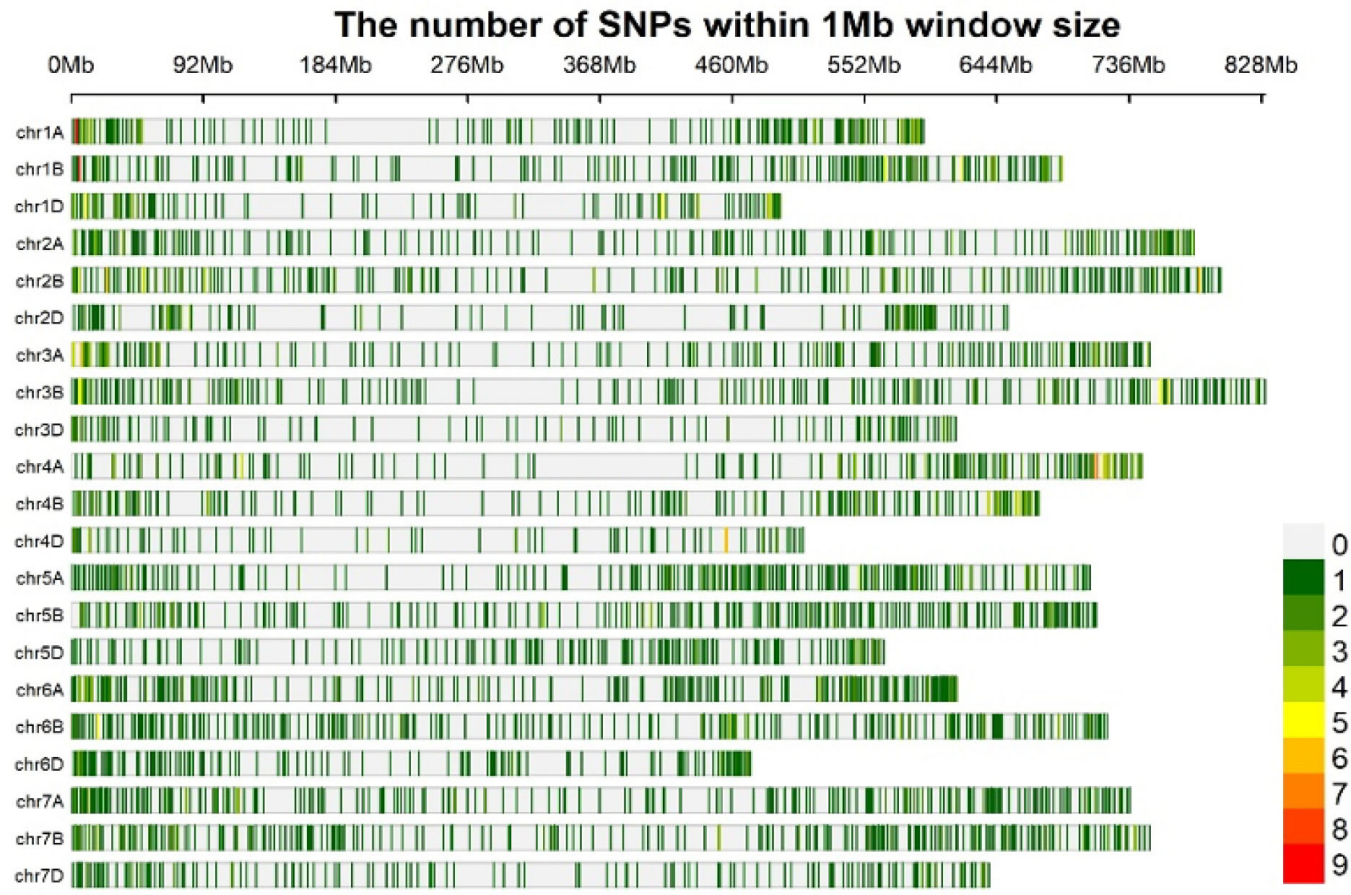
Based on their physical position, the genome-wide DArTSNP markers’ density for available markers within 3.9K DArTSNP array.

At the growth stage (GS), 12 of the plant, genomic DNA was isolated (Zadoks et al., 1974), and the modified Cetyl Trimethlyl Ammonium Bromide (CTAB) method was applied (Saghai-Maroof et al., 1984) for the isolation of DNA. Samples were subjected to snap freezing and grinding using liquid nitrogen. Following RNase treatment, aqueous phase separation was conducted at a centrifugal force of 13,000 rpm for 10 minutes at 4°C, utilizing a phenol:chloroform:isoamyl alcohol (25:24:1) solution. The sample was precipitated with chilled isopropanol, washed with 70% ethanol, and air-dried. After dissolving the pellet in nuclease-free water, the DNA concentration was measured at 260 nm using a Nano-Quant Infinite 200 spectrophotometer (TECAN, Salzburg, Austria). These 652 wheat accessions were genotyped using 3.9K DArTSNP genotyping (**Figure 1**). Calling and filtering of DArTSNP markers were performed in the R software using the dartR package by applying call rate, reproducibility, and heterozygosity with thresholds of 0.95, 0.5, and 0.7, respectively. The DArTSNP markers’ physical positions were referred to the sequences RefSeqv1.0 (https://excellenceinbreeding.org/toolbox/services/wheat-39k-mid-density-genotyping services-0).

### Genome-wide association study, linkage disequilibrium, and population structure

The STRUCTURE V 2.3.4 program was used for analyzing the population structure of 652 wheat genotypes (Pritchard et al., 2000). To determine the value of K, an admixture model was employed. The value of K indicates the number of subpopulations. For every K value between 1 and 10, 10 iterations were carried out for this analysis. A 10,000 burn-in length and three independent runs for each K value were additional parameters. The STRUCTURE HARVESTER software program (Earl and VonHoldt, 2012) was used to make this analysis easier. It generates K plots for the genotypes being studied and ensures that the most optimal K value is selected. The optimal number of sub-populations (K value) was measured using the procedure described by Evanno et al. (2005).

According to Bradbury et al. (2007), the TASSEL 5 software is a reliable tool for assessing LD. To determine the associations between genetic markers and phenotypes of interest, association mapping was performed in the Rmvp package of R software using the FarmCPU model (Liu et al., 2016), general linear models (GLMs), and mixed linear models (MLMs) (Yu et al., 2006). The single-locus MLM is traditionally the most used model for GWASs. It uses population structure (Q matrix) and kinship or family relatedness (K matrix) to control spurious associations (Zhang et al., 2005; VanRaden, 2008). However, this model was designed to test one marker at a time and is more likely to cause spurious associations (Wen et al., 2018). Multi-locus model FarmCPU is considered more efficient and reliable than single-locus models for mapping studies (Vikas et al., 2022). FarmCPU operates iteratively, using both fixed and random models, and incorporates significant SNPs as cofactors in each iteration to manage spurious associations without overfitting the model (Liu et al., 2016). As stripe rust resistance is based on a complex genetic structure and wheat has a high degree of LD, the significance threshold for finding MTAs was set at −log10(p-value) > 3. These association mapping results were visualized using quantile–quantile (Q–Q) plots and Manhattan plots. Significant markers observed were further subjected to *in silico* annotation (https://plants.ensembl.org/biomart/martview). Linking the significant markers with putative candidate genes was processed using a position-dependent strategy.

## Results

**Figure 2:**
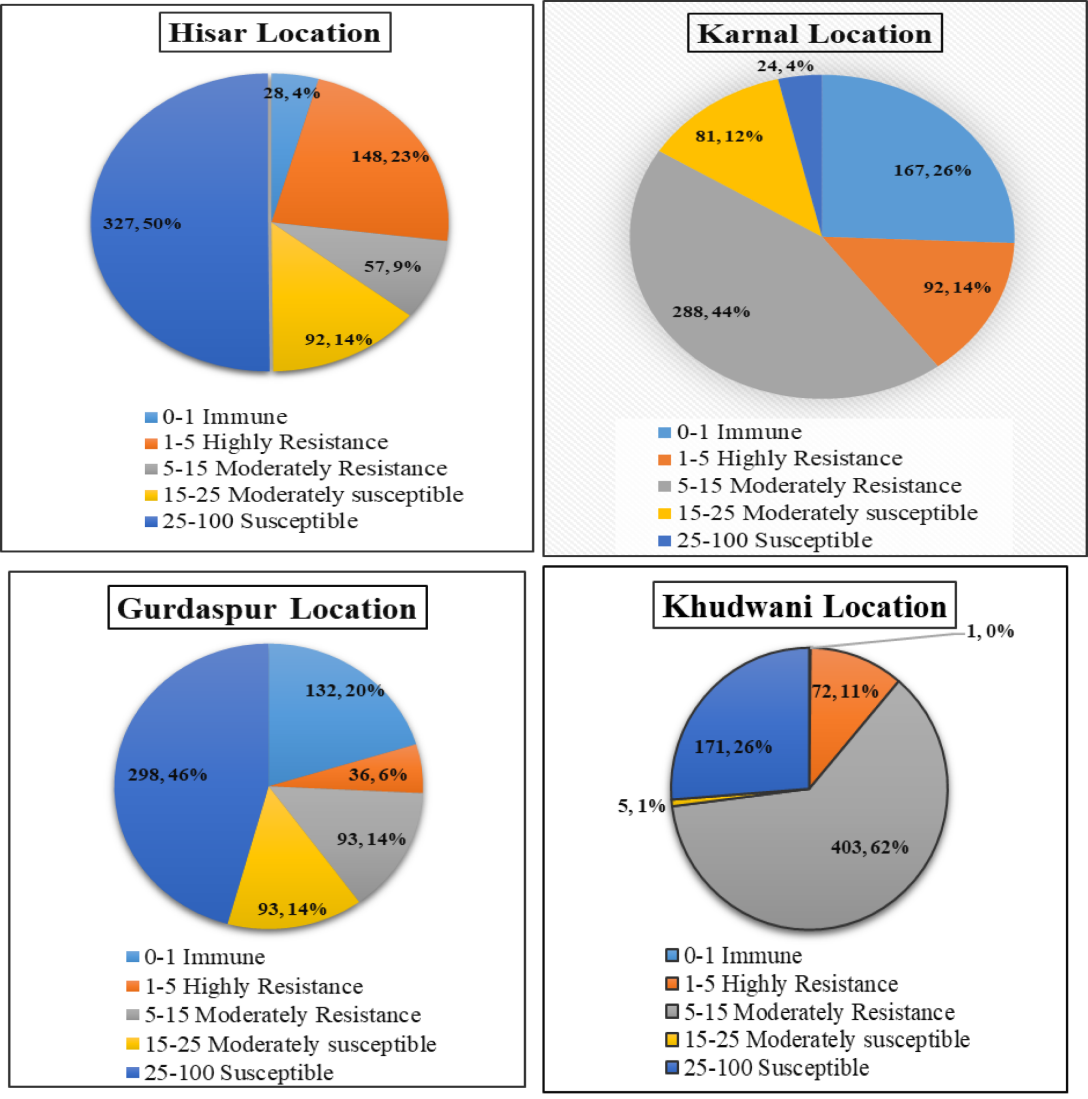
Pie chart representation of adult plant disease response against stripe rust (YR) of diverse wheat genotypes in corresponding environments (Hisar, Karnal, Gurdaspur, and Khudwani). The color legend on the right side of each pie chart represents the coefficient of infection (CI) score. The magnitude of arc length is directly proportional to the frequency of genotypes showing corresponding CI scores.

The genotypes for stripe rust resistance varied significantly across the locations, as shown by the ANOVA in **Table 2** (p-value ≤ 0.01). The responses of several genotypes to stripe rust are depicted in **Figure 2**, which makes it evident how many comparable reaction types there were. According to Roelfs et al. (1992), the main infection forms have been well documented, and the modified Cobb’s scale (Peterson et al., 1948) was used to calculate the severity of stripe rust as a percentage of the leaf surface area impacted. To ensure accuracy, field assessments were carried out three times. Final scores range from 80S (extremely susceptible) to 0 (immune). With a CI score between 0 and 1, a total of 148 of these genotypes exhibited a high resistance reaction at the Hisar location. Approximately 9% of the genotypes were MR, falling within the CI range of 5–15, and 327 genotypes (almost 50% of the genotypes) showed susceptible response with a CI range of 25–100. At the Karnal location, almost 44% of genotypes exhibited a moderately resistant-type reaction, with a CI value range of 5–15. Additionally, 167 genotypes (approximately 26%) showed an immune-type reaction. In the case of the Gurdaspur location, 298 genotypes (almost 46%) displayed susceptible responses with a CI value range of 25–100, and only 93 genotypes showed moderately susceptible reactions with a CI value range of 15–25. At the Khudwani location, 403 genotypes (62%) displayed moderately resistant-type reaction with a CI value range of 5–15, and 171 genotypes with a CI value range of 25–100 displayed susceptible reaction. Genotypes HI 8847, HI 8846, KBSN 17, KBSN 51, PBW 698, PBW 701, PBW 702, PBW 763, PBW 765, PBW 752, DBW 187, and WH 1270 showed highly resistant reactions across the locations. However, GABO, JW 3020, HD 2851, W 8627, BAXI 288-18, HYB-11, MACS 6145, RW 346, WR 544, and MONDHYA-32 showed highly susceptible reactions across the locations. There are some genotypes, viz., Lr-13sc and NP 721, that showed resistance at Karnal and Hisar but showed susceptible reaction at the Khudwani and Gurdaspur locations. Genotype NP 825 showed resistance at Khudwani but showed a highly susceptible reaction at Hisar, Karnal, and Gurdaspur. WH 291 showed a resistant reaction at Karnal and a highly susceptible reaction at Hisar, Gurdaspur, and Khudwani. The stripe rust data were visualized and considered for studying Pearson’s correlation in R statistical programming. Correlation plots for stripe rust describing the correlation of disease scores between different locations were created using the metan R package (**Supplementary Figure 1**). The highest correlation among disease scores was found between the Hisar and Gurdaspur locations, followed by Hisar and Khudwani. Also, some basic statistics for this data set were generated, which were indicative of variability for the trait and also the differential expression of disease across locations (**Supplementary Table 5**).

**Table 2:** Analysis of variance of 652 wheat genotypes for stripe rust resistance.

Source	*df*	Mean sum of squares							
		Hisar		Karnal		Gurdaspur		Khudwani	
Treatment (ignoring blocks)	654	608.94	**	80.79	**	899.39	**	457.12	**
Treatment: Check	2	25.00	^ns^	10.71	**	24.43	^ns^	5.57	^ns^
Treatment: Test	651	601.43	**	80.46	**	884.78	**	452.97	**
Treatment: Test vs. Check	1	6,663.20	**	434.79	**	12,156.64	**	4,061.42	**
Block (eliminating Treatments)	6	6.94	^ns^	1.43	^ns^	13.11	^ns^	12.10	^ns^
Residuals	12	11.89		1.05		17.04		13.24	

**Significant at (P< 0.01).

### Analysis of molecular variance and population structure analysis

**Figure 3:**
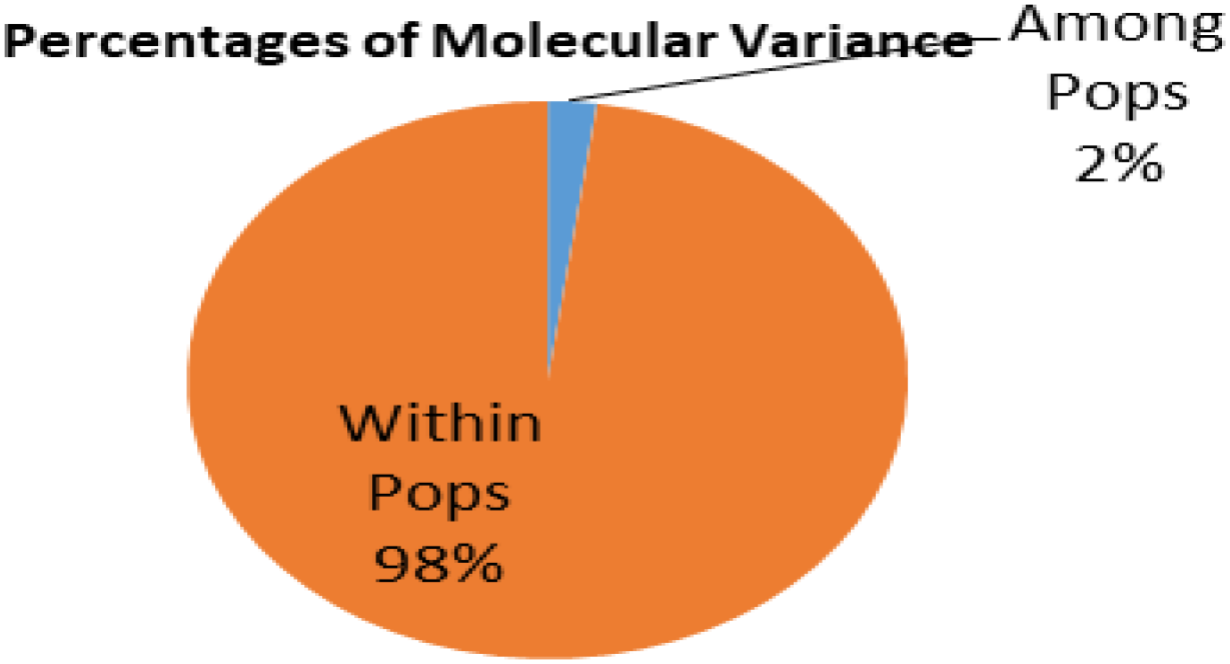
Analysis of molecular variance of subpopulation obtained through population structure analysis.

Using GenAlEx (version 6.5), Analysis of Molecular Variance (AMOVA) was performed, and it was found that the variations between groups identified by STRUCTURE analysis explained approximately 2% of the variation in the entire germplasm. Conversely, diversity within a group accounted for 98% of the variation (**Figure 3**). The population-wide fixation index (FST) value was 0.022, and it was deemed significant at p < 0.001. Variation among genotypes in the total population was particularly huge, despite pairwise FST values indicating that variation among subpopulations was lower. Data obtained from 1,938 DArTSNP markers were analyzed using the STRUCTURE software to study the population structure and for the identification of subgroups within the studied wheat genotypes.

**Figure 4:**
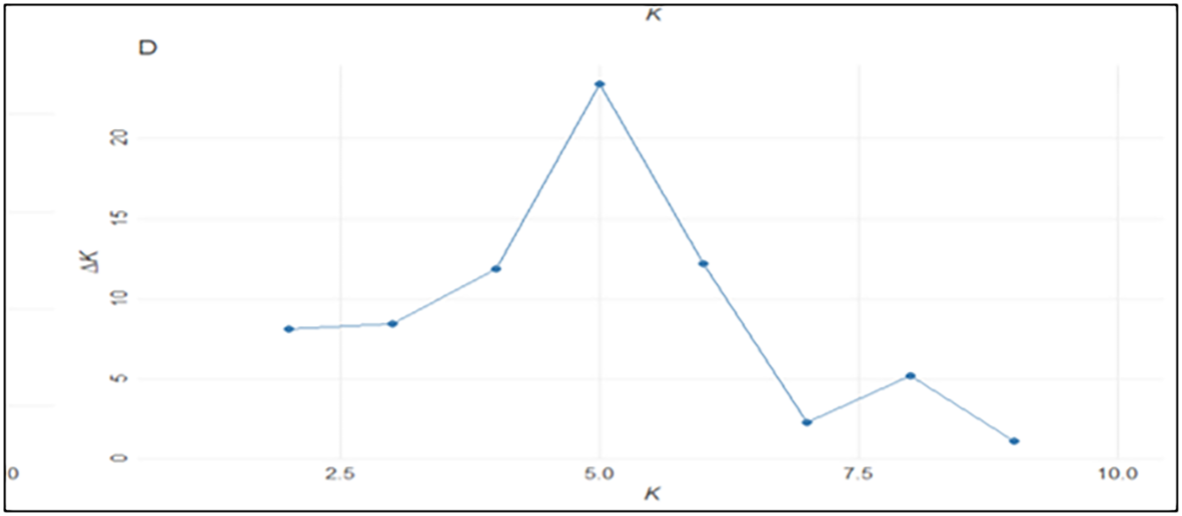
Population structure analysis: a ΔK for different numbers of subpopulations (K). Sharp peak was observed at K = 5 with maximum of ΔK.

**Figure 5:**
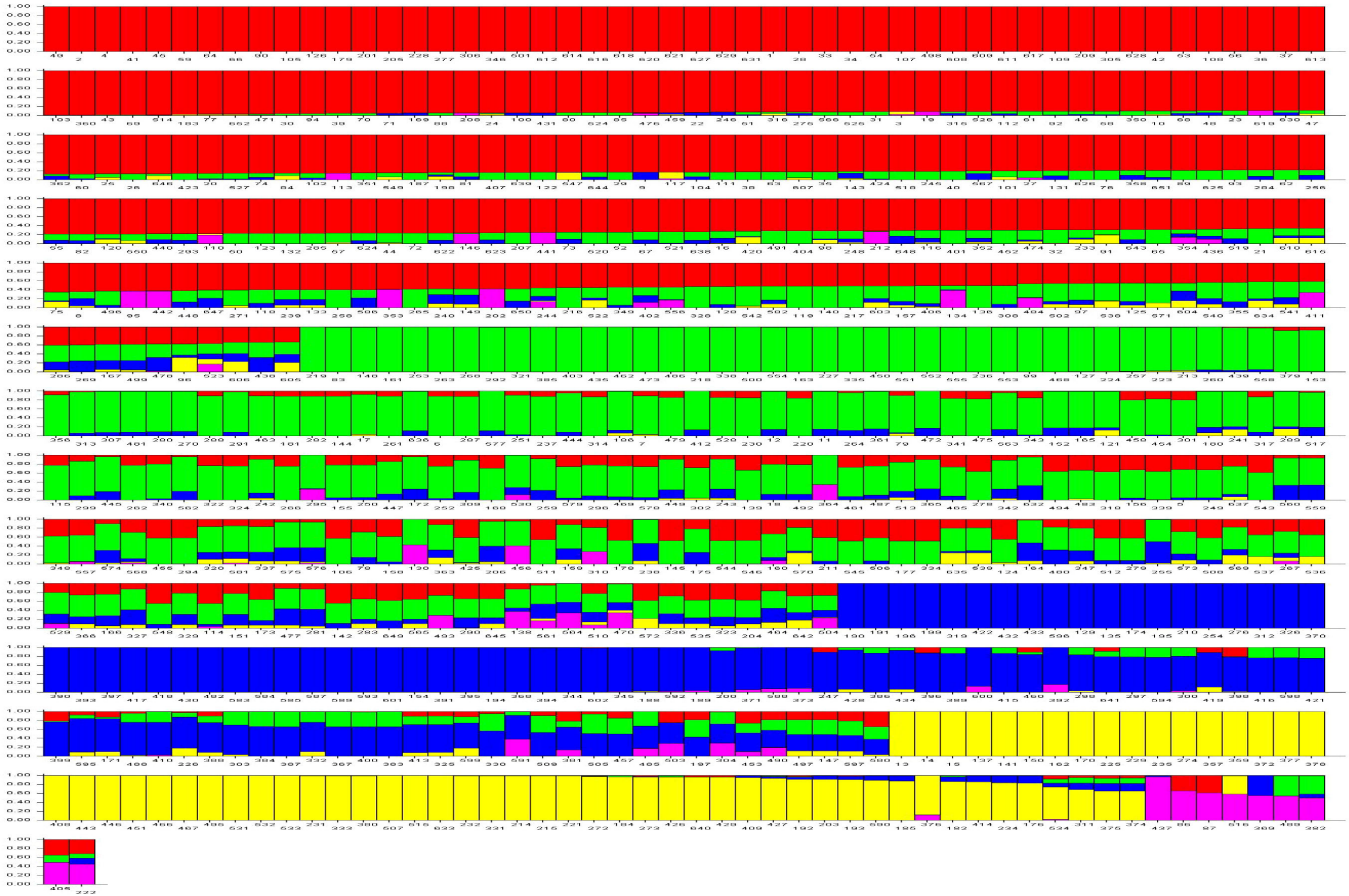
Population structure analysis: structure plot for 652 genotypes at K = 5, where each color represents one subpopulation, namely, SP1, SP2, SP3, SP4, and SP5.

The admixture model was utilized, with a membership probability threshold of 50% established to assign genotypes to specific clusters. The results of the STRUCTURE analysis showed that there were five major subgroups within the populations, based on the ΔK values for K ranging from 1 to 10 (**Figure 4**). Different colors were used to demonstrate these subgroups: 238 genotypes were found in the red cluster, 182 in the green cluster, 92 in the blue cluster, 60 in the yellow cluster, and seven in the purple cluster (**Figure 5**). The various numbers of genotypes in each category indicated that the wheat genotypes under study had varying degrees of genetic differentiation. Additionally, 72 genotypes were categorized as admixtures, representing their genetic contributions from various subgroups, because they did not exceed the threshold membership probability for inclusion in any of the clusters.

SP1 was mainly composed of varieties (~54%), advanced breeding lines (18.6%), and mutants (~10%), while varietal lines (~70.7%) and landraces (~16.5%) collectively made up SP2. The exact contributions of SP3, SP4, and SP5 are available in **Supplementary Table 2**. Additionally, genetic stocks contributed ~10% in SP1, 9.3% in SP2, 18.4% in SP3, and 8.4% in SP4; landraces were observed in SP3 (29.3%); and varietal lines, which were significant components of SP1 and SP2, also appeared in SP3 with contributions of 51%. SP4 and SP5 mainly composed 55.93% and 85.7%, respectively, of varieties. The mean of Infection Type (IT) scores for genotypes in subpopulations (SP1, SP3, SP4, and SP5) was higher than that of those in subpopulation two (SP2). This shows that in the current study, genotypes were more resistant in SP2 than in SP1, SP3, SP4, and SP5 to most of the pathotypes. Therefore, the influence of population structure on rust infection was considered a covariate for subsequent association analyses.

### Linkage disequilibrium

**Figure 6:**
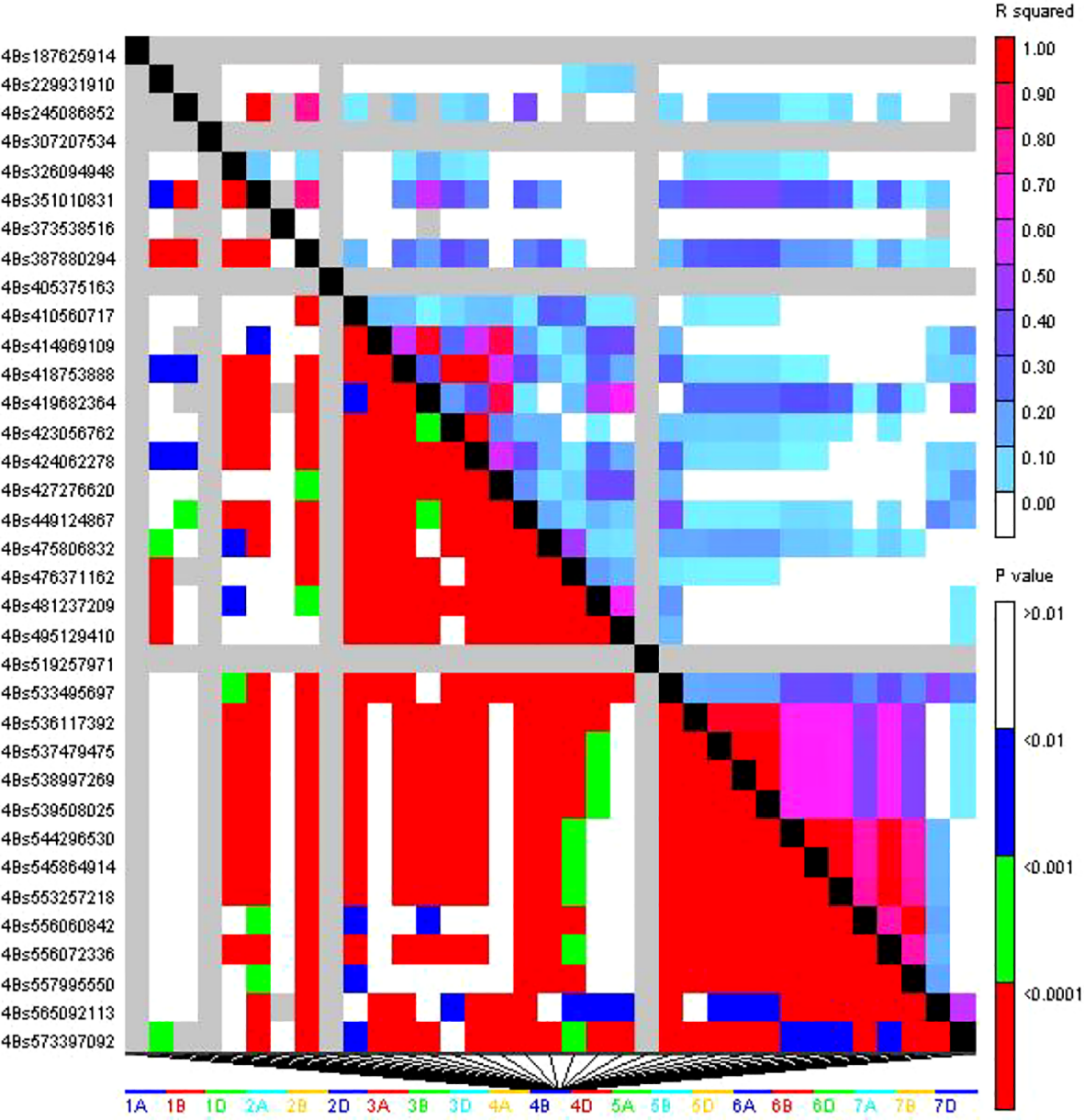
Triangle heat plot showing pairwise values of r^2^ and p for different locus pairs over different chromosomes of selected wheat genotype.

**Figure 7:**
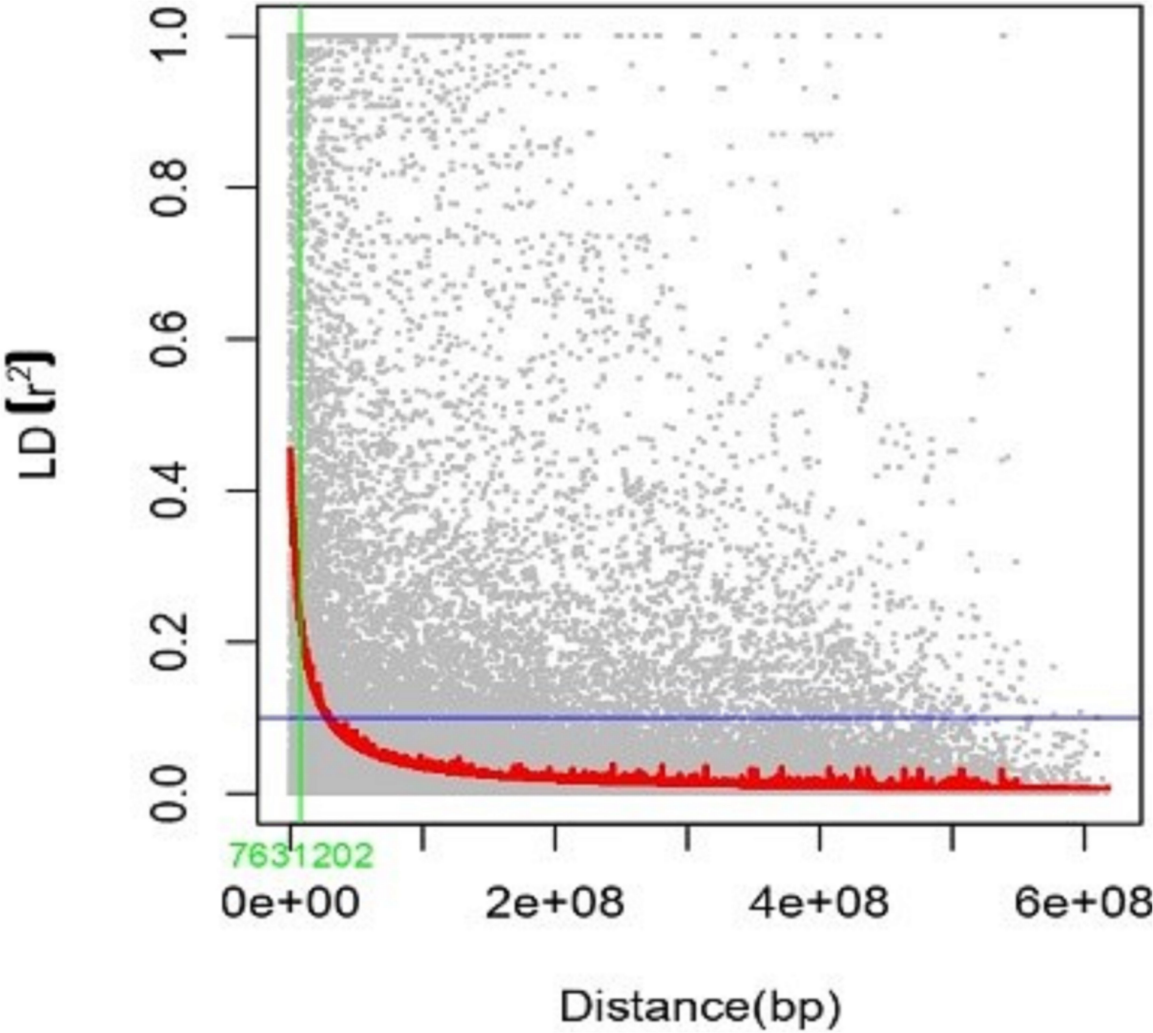
Scatter plot showing LD decay of pairwise SNP LD r2 value over the genetic distance between intra-chromosomal marker pairs.

LD was studied in 94,425 locus pairings using the 1,938 DArTSNP markers from the wheat genotype panel. Among the identified locus pairings, 15,052 pairs (36.56%) exhibited significant LD, which was observed at a threshold value of r^2^ > 0.05, indicating a non-random relationship between alleles at these loci (**Figure 6**). Significant LD was observed in 9,135 marker pairs when a stringent threshold of r^2^ ≥ 0.1 was applied. LD decayed at genetic distances of 7,631,202 bp for the whole genome (**Figure 7**). The map distance at which the fitted decay curve intersected with the critical r^2^ provided an estimate of QTL-CI. The estimated QTL-CI of 76,312,02 bp was observed in this study. LD beyond this critical value is considered to be caused by genetic linkage.

### Association mapping

A number of approaches, including GLM, MLM, and FarmCPU, were employed in order to find significant MTAs. The R software’s Rmvp package was used for all of these approaches. Different significant MTAs were identified based on location. The picture displays quantile–quantile (QQ) graphs between the observed and expected p-values of association using GLM, MLM, and FarmCPU models, revealing how effectively these models corresponded to all four locations.

**Figure 8:**
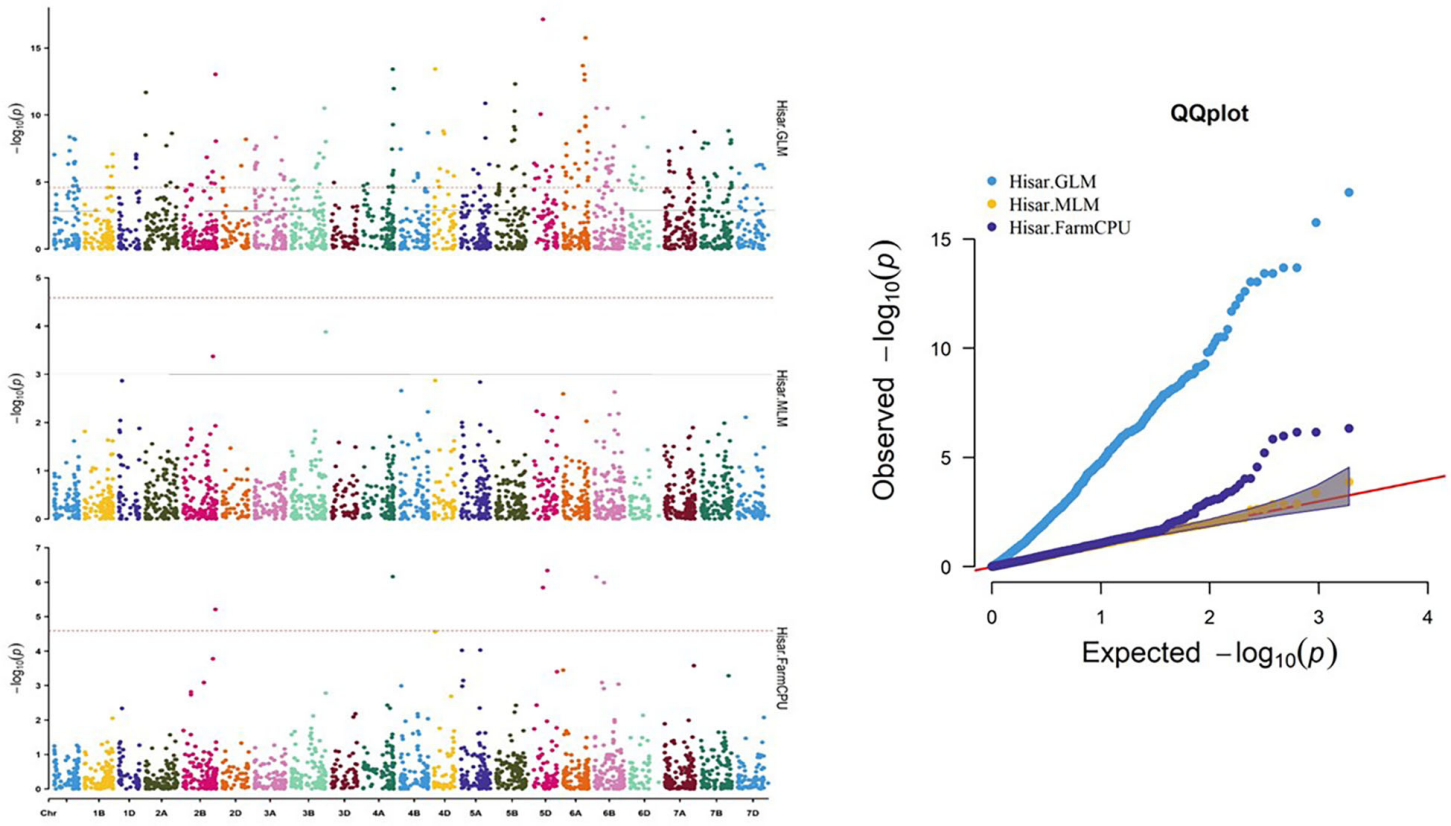
Manhattan and QQ plot for significance [−log10(p-values)] of the association of 1,938 DArTSNPs based on GLM, MLM, and FarmCPU located on 21 chromosomes with the adult plant disease responses at Hisar location. GLM, general linear model; MLM, mixed linear model.

**Figure 9:**
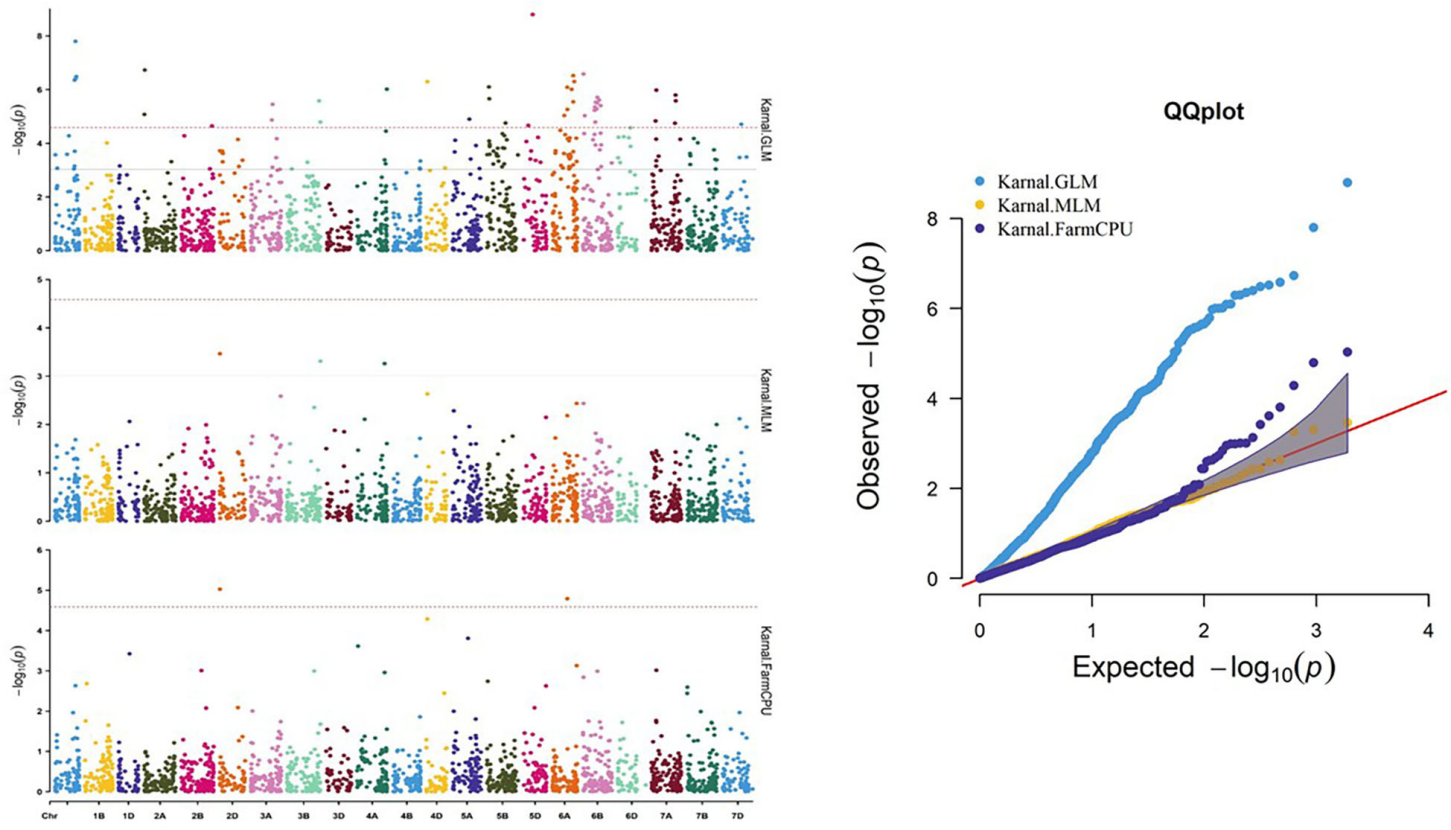
Manhattan and QQ plot for significance [−log10 (p-values)] of the association of 1,938 DArTSNPs based on GLM, MLM, and FarmCPU located on 21 chromosomes with the adult plant disease responses at Karnal location. GLM, general linear model; MLM, mixed linear model.

**Figure 10:**
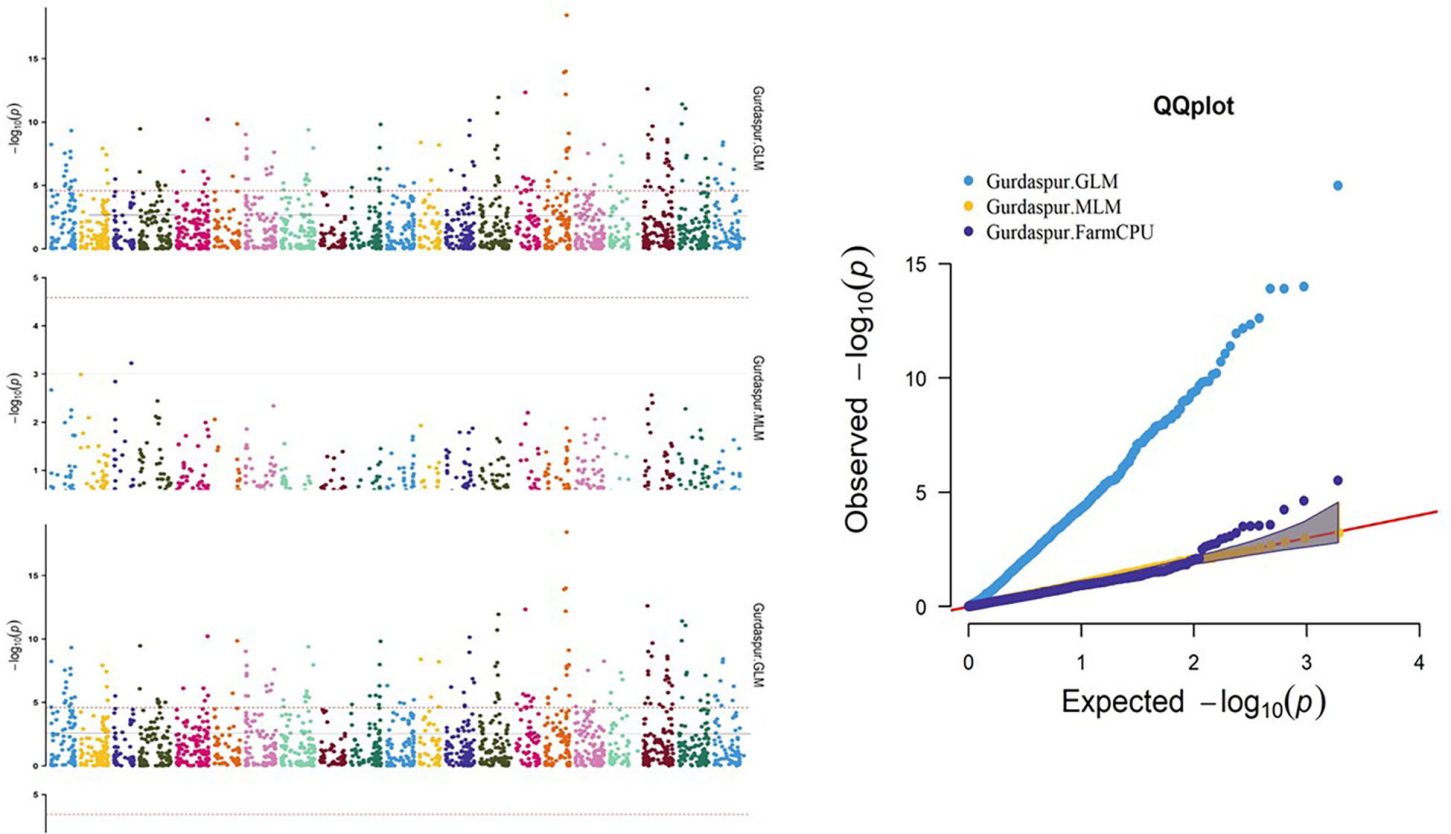
Manhattan and QQ plot for significance [−log10(p-values)] of the association of 1,938 DArTSNPs based on GLM, MLM, and FarmCPU located on 21 chromosomes with the adult plant disease responses at Gurdaspur location. GLM, general linear model; MLM, mixed linear model.

**Figure 11:**
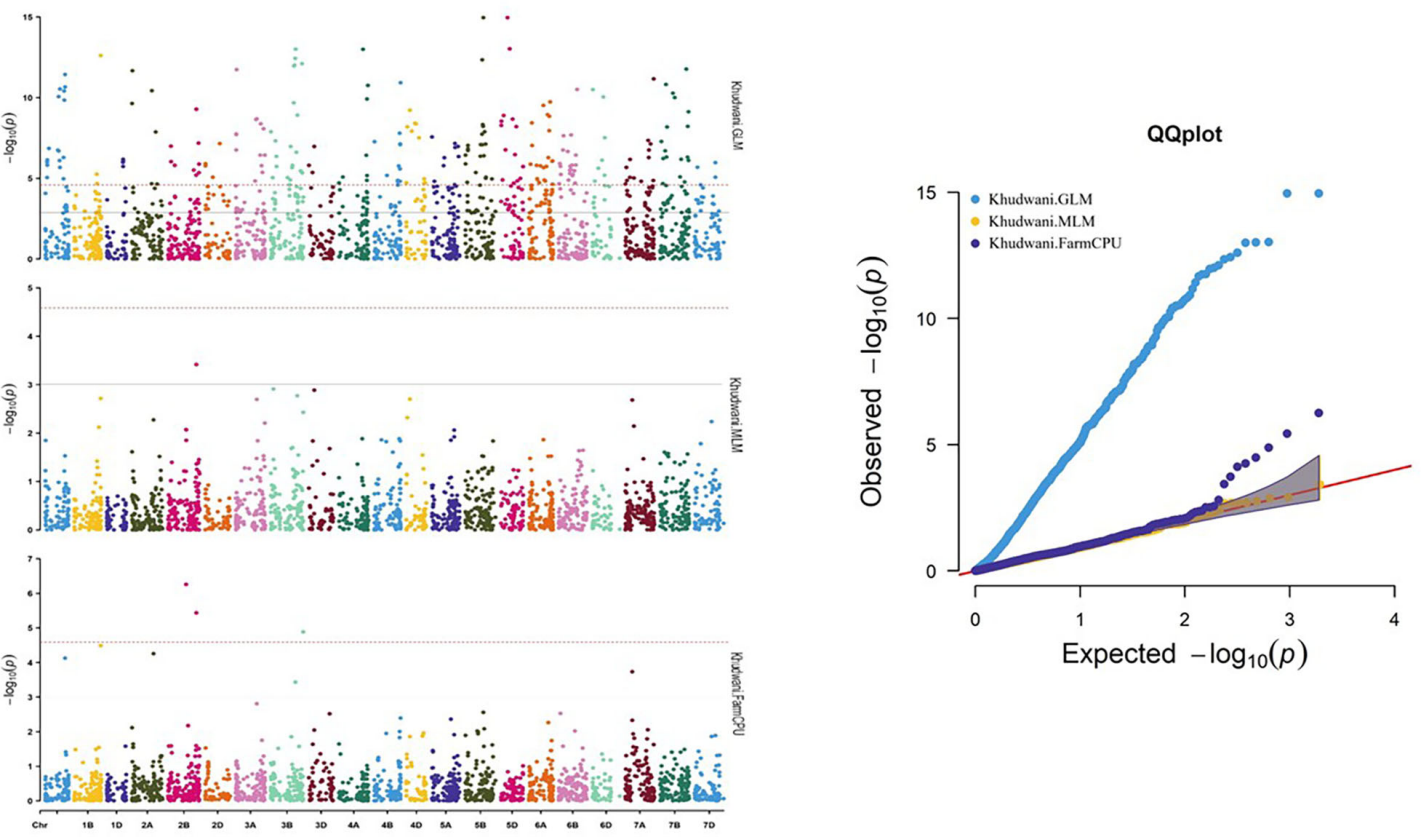
Manhattan and QQ plot for significance [−log10(p-values)] of the association of 1,938 DArTSNPs based on GLM, MLM, and FarmCPU located on 21 chromosomes with the adult plant disease responses at Khudwani location. GLM, general linear model; MLM, mixed linear model.

A total of 27 significant MTAs representing 14 chromosomes (Chr IA, 1B, 1D, 2B, 2D, 3B, 4A, 4D, 5A, 5D, 6A, 6B, 7A, and 7B) that were common in at least two models were found to be associated with stripe rust resistance at all four locations. At the Hisar location, out of the total identified MTAs, 14 MTAs were found that were common in at least two models to be associated with stripe rust resistance in wheat at −log10 p > 3 (**Figure 8**), same like in Karnal, Gurdaspur, and Khudwani, i.e., 7, 4, and 5, respectively (**Figure 9**). One MTA, TaDArTAG007561, was identified as common in three locations (**Figures 10**, **11**), i.e., Hisar, Karnal, and Khudwani; another MTA, TaDArTAG007677, was found common in two locations, i.e., Hisar and Karnal. This multi-model revealed that significant MTAs were environmentally specific, showing the presence of significant G × E interaction. The details of co-located QTLs with MTAs are given in **Table 3**. The emphasis was on the markers with important biological functions that have previously been validated and linked with response to disease resistance. The markers on 2B and 6B were found to correspond to leucine-rich repeat and serine/threonine-protein kinase-like domain disease resistance protein, while the SNPs identified on 6A corresponded to F-box and NB-ARC domain. Some regions also encoded an ABC transporter-like, ATP-binding domain located on 4A. Similarly, three different SNPs at chromosomes 1B, 3B, and 7A encoded receptor-like kinase proteins, which are also an important family of proteins with multiple functions, one of which is disease resistance. The many details of candidate genes are provided in **Supplementary Tables 3** and **Supplementary Table 4**.

**Table 3:** List of MTAs identified at different locations using different models. .

Location	DArT markers	SNP name	Chromosomal location	Position	p-Value	r^2^/PVE	GLM	MLM	FarmCPU	Colocalized QTLs	Reference
Karnal	TaDArTSNPSAG006173	D_contig17313_245	2D	9344681	0.000193	0.003556	P	P	P	–	–
Karnal	TaDArTSNPSAG007561	chr3B:830369163-830369463:chr3B_830369313	3B	8.3E+08	0.000153	0.003613	P	P	A	–	–
Karnal	TaDArTSNPSAG007622	chr4A:684967401-684967701:chr4A_684967551	4A	6.85E+08	0.000416	0.003066	P	P	A	*QYr.sgi-4A.1*	Agenbag et al., 2014
Karnal	TaDArTSNPSAG007769	chr6A:373995587-373995887:chr6A_373995737	6A	3.74E+08	8.22E−07	0.005706	P	A	P	*YrP10090*	Liu et al., 2021
Karnal	TaDArTSNPSAG007677	chr4D:26481348-26481648:chr4D_26481498	4D	26481498	0.000786	0.002001	P	A	P	–	–
Karnal	TaDArTSNPSAG006874	tplb0026o20_691	6A	6.07E+08	0.00048	0.002782	p	A	P	*QYr.ufs-6A*	Prins et al., 2011
Karnal	TaDArTSNPSAG007331	S7A_567030080	7A	5.67E+08	6.48E−07	0.005952	P	A	P	–	–
Hisar	TaDArTSNPSAG001883	S2B_716120567	2B	7.16E+08	0.000892	0.001949	A	P	P	*QYraq.cau-2B*, *QYrns.orz-2BL*	Guo et al., 2008; Vazquez et al., 2015
Hisar	TaDArTSNPSAG007561	chr3B:830369163-830369463:chr3B_830369313	3B	8.3E+08	0.000153	0.003613	P	P	A	–	–
Hisar	TaDArTSNPSAG000166	ALPb4A_773_SNP	4A	7.18E+08	1.07E−08	0.008659	P	A	P	–	–
Hisar	TaDArTSNPSAG004411	S6B_476413707	6B	4.76E+08	1.69E−06	0.005974	P	A	P	–	–
Hisar	TaDArTSNPSAG006947	Tdurum_contig4592_327	6B	5.72E+08	2.04E−07	0.006929	P	A	P	*QYr.nwafu-6BL*, *QYr.inra-6B*	Wu et al., 2018; Dedryver et al., 2009
Hisar	TaDArTSNPSAG009232	AX-94593432	5D	2.2E+08	4.68E−13	0.013579	P	A	P	–	–
Hisar	TaDArTSNPSAG000523	RAC875_rep_c116263_97	2B	7.75E+08	6.15E−11	0.009544	P	A	P	*QYrAvS.wgp-2BS*	Christiansen et al., 2006; Liu et al., 2020
Hisar	TaDArTSNPSAG007677	chr4D:26481348-26481648:chr4D_26481498	4D	26481498	0.000786	0.002001	P	A	P	–	–
Hisar	TaDArTSNPSAG003336	S5A_466029658	5A	4.66E+08	1.86E−05	0.003336	P	A	P	–	–
Hisar	TaDArTSNPSAG009350	AX-94979699	6A	5221698	0.000653	0.002996	P	A	P	*QYr.uga-6AS*	Hao et al., 2011
Hisar	TaDArTSNPSAG007213	BS00023166_51	7B	7.01E+08	9.07E−05	0.004002	P	A	P	–	–
Hisar	TaDArTSNPSAG003386	S5A_51793323	5A	51793323	0.000211	0.002731	P	A	P	–	–
Hisar	TaDArTSNPSAG001742	S2B_494862155	2B	4.95E+08	0.000604	0.002261	P	A	P	–	–
Hisar	TaDArTSNPSAG004467	S6B_572495855	6B	5.72E+08	0.000243	0.002822	P	A	P	–	–
Gurdaspur	TaDArTSNPSAG000018	Glu-B3fg_SNP	1B	5687022	0.000447	0.001419	P	P	P	*QYr.cim-1BS*	Zhang et al., 2022
Gurdaspur	TaDArTSNPSAG000073	TaFT3-D1	1D	4.3E+08	0.000413	0.003106	P	P	P	–	–
Gurdaspur	TaDArTSNPSAG001133	S1D_30446693	1D	30446693	3.15E−06	0.004769	P	A	P	–	–
Gurdaspur	TaDArTSNPSAG004338	S6B_229054418	6B	2.29E+08	5.14E−05	0.001602	P	A	P	–	–
Khudwani	TaDArTSNPSAG001891	S2B_717627572	2B	7.18E+08	5.44E−10	0.005772	P	P	P	*QYraq.cau-2B*, *QYrns.orz-2BL*	Guo et al., 2008; Vazquez et al., 2015
Khudwani	TaDArTSNPSAG007561	chr3B:830369163-830369463:chr3B_830369313	3B	8.3E+08	0.000153	0.003613	P	A	P	–	–
Khudwani	TaDArTSNPSAG005805	BS00066305_51	1B	6.76E+08	2.00E−13	0.010949	P	A	P	*QYr.sicau-1B.3*	Ma et al., 2019
										*QYrPI181410.wgp-1BL*	Liu et al., 2020
										*Qyr.gaas.1B.1*	Cheng et al., 2022
										*QYr.crc-1BL*	Rosa et al., 2019
										*QYr.spa-1B*	Bokore et al., 2017
										*QYrsn.nwafu-1BL*	Huang et al., 2021
										*QYr.sicau-1BL*	Wang et al., 2022
										*QYrdr.wgp-1BL.2*	Hou et al., 2015
										*QYrsk.wgp-1BL*	Liu et al., 2019
										*QYrsv.swust-1BL.2*	Zhou et al., 2021
										*QYr.cim-1BL*	Calvo-Salazar et al., 2015
Khudwani	TaDArTSNPSAG000206	S1A_500074551	1A	5E+08	5.99E−05	0.003392	P	A	P	–	–
Khudwani	TaDArTSNPSAG002689	S3B_629591171	3B	6.3E+08	0.000098	0.003964	P	A	P	–	–

MTAs, marker–trait associations; DArT, Diversity Arrays Technology; GLM, general linear model; MLM, mixed linear model; QTLs, quantitative trait loci.

## Discussion

The selection of highly diverse germplasm or genotypic panel is a key prerequisite for the success of any association mapping study. This diversity ensures comprehensive analysis and enhances the reliability of the findings. The statistical analysis (ANOVA) performed in the present study indicated significant variation among the genotypes. The variability for the trait and its expression across the locations can be further assessed by the basic statistics analyzed across the locations (**Supplementary Table 5**). Among all four regions used for screening rust resistance in the current wheat breeding program, e.g., Khudwani is considered a “yellow rust disease hotspot” in the Jammu and Kashmir regions. Also, Hisar and Karnal (Haryana) and Gurdaspur (Punjab) lie in the wheat rust-vulnerable belt. Khudwani (Jammu and Kashmir, Anantnag district) is at a higher altitude (~1,590–1,700 m above mean sea level) and lies in a Northern Hills Zone (NHZ). Gurdaspur (Punjab) lies in the sub‐mountainous/pre‐hill region (Kandi belt) and experiences earlier and more aggressive rust outbreaks because of its proximity to hilly inoculum sources. Hisar and Karnal (Haryana) are in the North‐Western Plain Zone (NWPZ), with lower-altitude flat plains. Epidemics occur but perhaps less routinely earlier compared to those in the foothills. The onset, severity, and timing of stripe rust may differ: during December, Khudwani and Gurdaspur may face earlier and higher disease pressure because of cooler/hill‐adjacent conditions; Hisar/Karnal, although susceptible, may have somewhat lower or delayed incidence depending on the season. In the current study, higher-than-average disease epidemics occur in the Gurdaspur (32.44) region, followed by Hisar (29.81166), Khudwani (19.00077), and Karnal (8.48). The possible region for lower infection at Hisar and Karnal is that the plains heat up earlier, possibly reducing the long favorable window for rust, and maybe agronomic/varietal conditions differ, giving Hisar and Karnal somewhat lower infection. The possible reason for lower infection at Khudwani is due to slightly cooler temperatures or shorter favorable windows for rust infection.

The above results assured that this study can confidently advance with the current panel of genotypes for association mapping.

### AMOVA, population structure, and LD

The AMOVA results showed that significant genetic diversity exists within subpopulations, with only 2% variation among them, and significant differences, as indicated by the partitioning value (p < 0.001), which is similar to an earlier study by Kumar et al. (2020). Frequent selection for economically significant features was shown by the significant variance within subpopulations. The *PhiPT* value (0.022) between subpopulations SP1, SP2, SP3, SP4, and SP5 was low, indicating little genetic differentiation among these subpopulations. This result is consistent with the AMOVA findings, which showed among-subpopulation variations accounting for only 2% of the total variation. Hence, genetic diversity analyses, including AMOVA, demonstrate that the genotypes used in this study possess ample diversity and could contribute to the development program for stripe rust resistance.

Based on the highest likelihood value of ΔK, which was identified at K = 5, the genotypes were separated into five subgroups, which were followed by K = 6 and K = 4. The relatively small number of significant clusters may be due to the close ancestral relationships and similar geographical origins of the majority of genotypes in this study. The highest likelihood score was reported at ΔK = 2 by Kumar et al. (2020), which resulted in the division of large germplasm into two clusters, whereas Zegeye et al. (2014); Chen et al. (2020), El-Messoadi et al. (2022), and El-Messoadi et al (2023) noted likelihood scores at ΔK = 5 or higher, which caused the clustering of genotype panels accordingly.

The results showed that there was significant linkage for 9,135 marker pairs (21.46%) at r^2^ ≥ 0.1 and for 15,056 marker pairs (36.56%) at r^2^ ≥ 0.05. The critical value of r^2^ was 0.16, which was comparable to the findings of other studies by Zegeye et al. (2014); El Hanafi et al. (2021), and El-Messoadi et al (2023). Triangle plots of pairwise LD may indicate larger LD blocks due to selection pressure on genotypes or varieties within breeding programs targeted at specific desirable traits.

### Association mapping and marker trait associations

Three distinct models—GLM, MLM, and FarmCPU—were used to predict the relationship between stripe rust resistance and DArTSNP markers. Several studies have shown that applying two or more distinct models to find favorable MTAs in wheat is an effective method to determine which model best fits the association mapping data (Tene et al., 2022; Abou-Zeid and Mourad, 2021; Habib et al., 2020). Comparative results from these models enhance the findings and reduce the potential for false associations. The LD results for 1,938 DArTSNP markers encouraged further association mapping. Notably, El-Messoadi et al (2022) previously conducted association mapping with 5,176 polymorphic markers using an MLM. Diversity Arrays Technology (DArT) markers on 426 synthetic bread wheat genotypes revealed three DArT markers on chromosomes 1B, 2B, and 7B that were significantly associated with resistance to stripe rust. Similarly, a comparable study conducted by El Hanafi et al. (2021) in 2014/2015 identified 23 markers that were strongly associated with adult plant resistance on chromosomes 2A, 2B, 2D, and 7B.

Thirty significant MTAs for stripe rust resistance (which were common to at least two models) were considered promising MTAs. Two markers were also found common across the locations, i.e., one in three locations and another marker in two locations, so a total of 27 genomic regions were identified. Comparative mapping with previous GWASs and QTL mapping studies for stripe rust resistance revealed that nine SNPs were co-localized within genomic regions of previously identified *Yr* genes/QTL, while 18 were located in regions not previously known to harbor stripe rust resistance genes/loci and thus were considered novel. Most of the highly significant MTAs were found on chromosomes 2B and 6B (**Table 3**). A low value of r^2^/Proportion of Variance Explained (PVE) for stripe rust in this study may be due to the polygenic nature of resistance and also due to multi-environmental interactions between the pathogen, host, and environmental factors. Stripe rust, being a complex trait, may often lead to a small fraction of phenotypic variance due to the small effect sizes of individual loci.

Numerous *Yr* genes and QTL have been mapped on chromosome 1B, including *Yr9*, *Yr10*, *Yr15*, *Yr24/Yr26/YrCh42*, *Yr64*, *Yr65*, *YrTr1*, *YrAlp*, *YrH52*, *YrH122*, *YrL693*, *YrC142*, *YrMY41*, *QYr.cau-1BS*, *QYrco.wpg-1BS.1*, and *QYrco.wpg-1BS.2* (Feng et al., 2018). TaDArTAG000018 with SNP name Glu-B3fg_SNP was found to be associated with QYr.cim-1BS (Zhang et al., 2022), and TaDArTAG005805 with SNP name BS00066305_51 was found within the genomic regions of QYr.sicau-1B.3 (Ma et al., 2019), QYrPI181410.wgp-1BL (Liu et al., 2020b), QYr.crc-1BL (Rosa et al., 2019), QYr.spa-1B (Bokore et al., 2017), QYrsn.nwafu-1BL (Huang et al., 2021), QYr.sicau-1BL (Wang et al., 2022), QYrdr.wgp-1BL.2 (Hou et al., 2015), QYrsk.wgp-1BL, QYrsv.swust-1BL.2 (Zhou et al., 2021), and QYr.cim-1BL (Calvo-Salazar et al., 2015). BLAST analysis of these markers identified candidate genes TraesCS1B03G0005800, TraesCS1B03G0007200, and TraesCS1B03G0008000, which encode for NB-ARC, leucine-rich repeat, and serine/threonine-protein kinase domains, which play a major role in disease resistance in plants (Van Ooijen et al., 2008; Annan and Huang, 2023; Gou et al., 2015; Klymiuk et al., 2018; Kumar et al., 2020). This is a large family of resistance genes that are often directly involved in the identification of pathogen effectors and form a major component of the wheat plant immune system.

On chromosome 2B, many genes, *yr31* (Yang et al., 2013), *yr7* (Marchal et al., 2018), *yr53* (Xu et al., 2013), and *yr41* (Luo et al., 2005), have been mapped for stripe rust resistance. MTA TaDArTAG000523 with SNP name RAC875_rep_c116263_97 was found within the genomic regions of the already identified QTLs *QYraq.cau-2BL* (Guo et al., 2008) and *QYrns.orz-2BL* (Vazquez et al., 2015). BLAST analysis of these markers identified candidate genes TraesCS2B03G1451500, TraesCS2B03G1444300, and TraesCS2B03G1449600, which encode for leucine-rich repeat, protein kinase domain serine/threonine-protein kinase domains (Klymiuk et al., 2018; Gou et al., 2015), which play a major role in disease resistance in plants. The kinases are cell-surface receptors and assist in detecting the pathogen and initiating the plant’s defense response. These proteins play a role during the seedling stage under higher temperatures. MTAs TaDArTAG001883, TaDArTAG001891 with SNP name *S2B_716120567*, and *S2B_717627572* were found to be associated with already identified QTL *QYrAvS.wgp-2BS* (Liu et al., 2020). BLAST analysis of these markers identified candidate genes TraesCS2B03G1299100, TraesCS2B03G1284400, and TraesCS2B03G1289400, which encode for F-box domain, protein kinase domain, and Zinc finger domain. Zinc finger protein functions as a negative regulator of programmed cell death and contributes to wheat resistance to yellow rust (Guo et al., 2013). One DArT marker, TaDArTAG001742 with SNP name *S2B_*494862155, was found far away from any QTL and already identified genes, hence tagging novel genomic regions. BLAST analysis and anchoring the flanking marker allowed the identification of the candidate gene TraesCS2B03G0888600 that encodes an ABC transporter-like, ATP-binding domain, which plays a crucial role in gene resistance (Krattinger et al., 2019). The stripe rust genes holding resistance during the adult plant stage provide broad-spectrum resistance, as they activate the general defense mechanisms.

MTA on chromosome 6B TaDArTAG004411 and TaDArTAG006947 found within two already identified QTLs *QYr.nwafu-6BL* (Wu et al., 2018) and *QYr.inra-6B* (Dedryver et al., 2009) indicated their association with stripe rust resistance. However, two other MTAs on this chromosome—TaDArTAG004338 and TaDArTAG004467—were identified away from any previously identified genes or QTLs, proving their novelty. *In silico* gene annotation helps in the identification of candidate genes present in these genomic regions. Candidate genes TraesCS6B03G0912300 and TraesCS6B03G0914600 present in this genomic region encode serine/threonine-protein kinase domain and leucine-rich repeat. Both these domains play a major role in disease resistance against stripe rust (Gou et al., 2015; Annan and Huang, 2023). On chromosome 6A, three MTAs were identified, with all MTAs found within the genomic regions of already identified QTLs on 6A *QYr.uga-6AS* (Hao et al., 2011), *YrP10090* (Liu et al., 2021), and *QYr.ufs-6A* (Prins et al., 2011). Candidate genes identified within these genomic regions encode NB-ARC, leucine-rich repeat, disease resistance protein, plants, and F-box domain; all have disease resistance properties (Annan and Huang, 2023). Two MTAs were identified on chromosome 1D—TaDArTAG000073 and TaDArTAG001133—with SNP names TaFT3-D1 and S1D_30446693, respectively. Both of these MTAs were identified far away from previously identified genes or QTLs, showing their novelty. *In silico* gene annotation led to the identification of candidate genes. The identified candidate genes TraesCS1D03G0807200 and TraesCS1D03G0805600 encode leucine-rich repeat domain superfamily (Annan and Huang, 2023) and Zinc finger, PHD-type domain, which plays a defense role against plant disease.

Two MTAs were identified on chromosome 4A (TaDArTAG000166 and TaDArTAG007622) with SNP name *ALPb4A_773_SNP* and *chr4A:684967401-684967701:chr4A_684967551.* TaDArTAG000166 marker was found within the genomic region of QTL *QYr.sgi-4A.1* (Agenbag et al., 2014). TaDArTAG007622 marker was located very close to the already identified *yr60* gene (Herrera-Foessel et al., 2015). The candidate gene identified in the genomic region encodes a protein kinase domain, leucine-rich repeat domain, and NB-ARC domain. All these domains help in developing resistance in the plant against stripe rust. Two MTAs were identified on chromosome 5A, i.e., TaDArTAG003336 and TaDArTAG003386. TaDArTAG003336 was associated with QTL *QYr.hebau-5AL* (Gebrewahid et al., 2020), showing its role in disease resistance. TaDArTAG003386 was not found close to any loci, proving its novelty. The candidate genes (TraesCS5A03G0129300, TraesCS5A03G0129600, and TraesCS5A03G0130800) identified in the protein kinase domain, serine/threonine-protein kinase, active site, and F-box-like domain superfamily play a role in disease resistance (Chen, 2020; Brueggeman et al., 2006). MTAs identified on chromosome 3B, i.e., TaDArTAG002689 and TaDArTAG007561, were not found within the genomic regions of any genes or QTLs, showing their novelty. Candidate genes TraesCS3B03G0972900, TraesCS3B03G0973500, and TraesCS3B03G0973500 identified in their genomic regions encode domains, i.e., Zinc finger C2H2-type, leucine-rich repeat domain superfamily, and protein kinase domain, which play a defense role against plant disease (Guo et al., 2013; Annan and Huang, 2023; Chen, 2020).

## Conclusion

Association mapping based on LD was successfully conducted in 652 wheat genotypes using 1,938 polymorphic DArTSNP markers. Extensive phenotyping across various locations revealed that a significant variation exists in stripe rust resistance among the genotypes. The molecular variation detected using the 1,938 polymorphic markers enables the categorization of all genotypes into five distinct clusters. Utilizing the three most effective methods for association mapping, this study identified a robust set of markers associated with stripe rust resistance. All 30 promising MTAs identified in this study corroborate findings from previous research. These identified MTAs will be instrumental for marker-assisted selection, facilitating the development of superior haplotypes with strong positive effects for stripe rust resistance and paving the way for further research on the underlying causal genes’ fine mapping and cloning. The key putative candidate genes may be the important candidates for further validation and gene cloning experiments.

## Data Availability

All relevant data is contained within the article: The original contributions presented in the study are included in the article/**Supplementary Material**, further inquiries can be directed to the corresponding author.
