# Supplementary figures and images for: Genome-wide association studies for identification of stripe rust resistance loci in diverse wheat genotypes
Source: Front Plant Sci. 2025 Dec 9;16:1687331. doi: 10.3389/fpls.2025.1687331 (PMC12746750; doi:10.3389/fpls.2025.1687331)

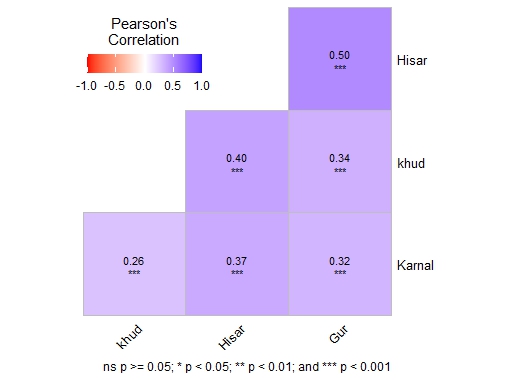

Supplement: Supplementary file 1 [file Image1.jpeg]
