# Supplementary material for: Genome-wide association studies for identification of stripe rust resistance loci in diverse wheat genotypes
Source: Front Plant Sci. 2025 Dec 9;16:1687331. doi: 10.3389/fpls.2025.1687331 (PMC12746750; doi:10.3389/fpls.2025.1687331)
Supplement: Supplementary file 2 [file Table1.doc]

**Supplementary Table 1 List of germplasm lines used in the current study**

| Sr. no | Name of Genotype | Type of Genotype |
| --- | --- | --- |
| 1 | HD 3436 | ABL |
| 2 | HD 2967 | V |
| 3 | HD 3437 | ABL |
| 4 | HD 3406 | V |
| 5 | HD 3438 | ABL |
| 6 | HD 2932 | V |
| 7 | HD 3439 | ABL |
| 8 | HD 3440 | ABL |
| 9 | HD 2733 | V |
| 10 | HD 3411 | V |
| 11 | PBW 901 | ABL |
| 12 | PBW 175 | V |
| 13 | HI 8846 | V |
| 14 | HI 8498 | V |
| 15 | HI 8847 | V |
| 16 | PBW 902 | ABL |
| 17 | HD 3407 | ABL |
| 18 | KBSN 1 | ABL |
| 19 | KBSN 3 | ABL |
| 20 | KBSN 4 | ABL |
| 21 | KBSN 5 | ABL |
| 22 | KBSN 6 | ABL |
| 23 | KBSN 7 | ABL |
| 24 | KBSN 8 | ABL |
| 25 | KBSN 9 | ABL |
| 26 | KBSN 10 | ABL |
| 27 | KBSN 11 | ABL |
| 28 | KBSN 12 | ABL |
| 29 | KBSN 13 | ABL |
| 30 | KBSN 14 | ABL |
| 31 | KBSN 15 | ABL |
| 32 | KBSN 16 | ABL |
| 33 | KBSN 17 | ABL |
| 34 | KBSN 18 | ABL |
| 35 | KBSN 19 | ABL |
| 36 | KBSN 20 | ABL |
| 37 | KBSN 21 | ABL |
| 38 | KBSN 22 | ABL |
| 39 | KBSN 23 | ABL |
| 40 | KBSN 24 | ABL |
| 41 | KBSN 25 | ABL |
| 42 | KBSN 26 | ABL |
| 43 | KBSN 27 | ABL |
| 44 | KBSN 28 | ABL |
| 45 | KBSN 29 | ABL |
| 46 | KBSN 30 | ABL |
| 47 | KBSN 31 | ABL |
| 48 | KBSN 32 | ABL |
| 49 | KBSN 33 | ABL |
| 50 | KBSN 34 | ABL |
| 51 | KBSN 35 | ABL |
| 52 | KBSN 36 | ABL |
| 53 | KBSN 37 | ABL |
| 54 | KBSN 38 | ABL |
| 55 | KBSN 39 | ABL |
| 56 | KBSN 40 | ABL |
| 57 | KBSN 41 | V |
| 58 | KBSN 42 | V |
| 59 | KBSN 43 | V |
| 60 | KBSN 44 | V |
| 61 | KBSN 45 | V |
| 62 | KBSN 46 | V |
| 63 | KBSN 47 | V |
| 64 | KBSN 48 | V |
| 65 | KBSN 49 | V |
| 66 | KBSN 50 | V |
| 67 | KBSN 51 | V |
| 68 | KBSN 52 | V |
| 69 | KBSN 53 | V |
| 70 | KBSN 54 | V |
| 71 | KBSN 55 | V |
| 72 | KBSN 56 | V |
| 73 | DBW 332 | V |
| 74 | DBW 327 | V |
| 75 | DBW 296 | V |
| 76 | HUW 838 | V |
| 77 | JKW 261 | V |
| 78 | GW 513 | V |
| 79 | HI 1636 | V |
| 80 | WH 1270 | V |
| 81 | HD 3298 | V |
| 82 | HD 3293 | V |
| 83 | CG 1029 | V |
| 84 | DBW 252 | V |
| 85 | DBW 222 | V |
| 86 | PBW 723 | V |
| 87 | PBW 771 | V |
| 88 | HI 1621 | V |
| 89 | HI 1628 | V |
| 90 | DBW 187 | V |
| 91 | HD 3226 | V |
| 92 | PBW 752 | V |
| 93 | HD 3237 | V |
| 94 | DBW 173 | V |
| 95 | HI 1612 | V |
| 96 | K 1317 | V |
| 97 | DBW 168 | V |
| 98 | HI 1605 | V |
| 99 | RAJ 4238 | V |
| 100 | HS 562 | V |
| 101 | DBW 107 | V |
| 102 | HD 3118 | V |
| 103 | DBW 110 | V |
| 104 | DBW 93 | V |
| 105 | DBW 88 | V |
| 106 | K 1006 | V |
| 107 | WH 1105 | V |
| 108 | DBW 71 | V |
| 109 | HPW 349 | V |
| 110 | HD 3043 | V |
| 111 | PBW 644 | V |
| 112 | KRL 210 | V |
| 113 | DPW 621-50 | V |
| 114 | HI 1563 | V |
| 115 | HD 2985 | V |
| 116 | HS 507 | V |
| 117 | DBW 39 | V |
| 118 | VL 907 | V |
| 119 | MACS 6222 | V |
| 120 | CBW 38 | V |
| 121 | HS 490 | V |
| 122 | PBW 550 | V |
| 123 | HPW 251 | V |
| 124 | VL 892 | V |
| 125 | WH 1021 | V |
| 126 | DBW 17 | V |
| 127 | GW 366 | V |
| 128 | DBW 16 | V |
| 129 | HD 2888 | V |
| 130 | HD 2864 | V |
| 131 | PBW 502 | V |
| 132 | VL 829 | V |
| 133 | HS 420 | V |
| 134 | DBW 14 | V |
| 135 | HI 1500 | V |
| 136 | GW 322 | V |
| 137 | HD 4672 | V |
| 138 | UP 2425 | V |
| 139 | HUW 468 | V |
| 140 | GW 273 | V |
| 141 | MACS 2846 | V |
| 142 | PBW 373 | V |
| 143 | VL 738 | V |
| 144 | RAJ 3765 | V |
| 145 | K 9107 | V |
| 146 | UP 2338 | V |
| 147 | WH 542 | V |
| 148 | GW 173 | V |
| 149 | HS 277 | V |
| 150 | MACS 2496 | V |
| 151 | KRL 1-4 | V |
| 152 | HD 2428 | V |
| 153 | RAJ 3077 | V |
| 154 | K 8027 | V |
| 155 | HI 1077 | V |
| 156 | HI 977 | V |
| 157 | MACS 1967 | V |
| 158 | HUW 234 | V |
| 159 | VL 616 | V |
| 160 | HD 2329 | V |
| 161 | HD 2285 | V |
| 162 | RAJ 1555 | V |
| 163 | RAJ 1482 | V |
| 164 | LOK 1 | V |
| 165 | HD 2189 | V |
| 166 | UP 262 | V |
| 167 | WH 147 | V |
| 168 | WL 711 | V |
| 169 | HD 2009 | V |
| 170 | RAJ 911 | V |
| 171 | NI 5439 | V |
| 172 | HD 1982 | V |
| 173 | HD 1941 | V |
| 174 | KHARCHIA 65 | V |
| 175 | SONALIKA | V |
| 176 | Ajanta | V |
| 177 | Amrut | V |
| 178 | 8A | LR |
| 179 | A 28 | LR |
| 180 | A 9-30-1 | LR |
| 181 | A 90 | LR |
| 182 | A 115 | LR |
| 183 | A 206 | LR |
| 184 | AKW-381 | LR |
| 185 | A 624 | LR |
| 186 | AKW 1071(PURNA) | LR |
| 187 | AKAW 4627 | LR |
| 188 | AKDW 2997-16 | LR |
| 189 | AKAW 3722 | LR |
| 190 | BIRSA GEHUN 2 | LR |
| 191 | BIJAGA YELLOW | LR |
| 192 | BIJAGA RED | LR |
| 193 | BW II (PURBALI) | LR |
| 194 | BIRSA GEHUN 3 | V |
| 195 | BA*I 288-18 | LR |
| 196 | CHHOTI LERMA | V |
| 197 | COW(W)1 | V |
| 198 | C 281 | V |
| 199 | C 285 | V |
| 200 | C 286 | V |
| 201 | C 306 | V |
| 202 | C 518 | V |
| 203 | C 591 | V |
| 204 | CPAN 1676(ROHINI) | V |
| 205 | CPAN 1796 | V |
| 206 | CPAN 3004(SANGAM) | V |
| 207 | DURGAPURA 65 | V |
| 208 | DWR 16 (KEERTHI) | V |
| 209 | DWR 39 (PRAGATI) | V |
| 210 | D 134 | V |
| 211 | DWR 137 | V |
| 212 | DWR 195 (ANURADHA) | V |
| 213 | DWR 225 | V |
| 214 | DWR 185 | V |
| 215 | DL 153-2 (KUNDAN) | V |
| 216 | DL 788-2 (VIDISHA) | V |
| 217 | DL 784-3 (VAISHALI) | V |
| 218 | DL 803-3 (KANCHAN) | V |
| 219 | DWR 1006 | V |
| 220 | DWL 5023 | V |
| 221 | FEDERATION | Exotic line |
| 222 | GW 496 | V |
| 223 | GW 503 | V |
| 224 | GULAB | V |
| 225 | GW 1139 | V |
| 226 | GW 1189 | V |
| 227 | GW 1 | LR |
| 228 | GW 2 | LR |
| 229 | GW 10 | LR |
| 230 | GW 11 | LR |
| 231 | GW 18 | LR |
| 232 | GW 40 | LR |
| 233 | GW 89 | LR |
| 234 | GW 120 | LR |
| 235 | GW 190 | LR |
| 236 | HI 1544 | V |
| 237 | HI 7483 | LR |
| 238 | HI 385 (MUKTA) | V |
| 239 | HI 617 (SUJATA) | V |
| 240 | HI 784 (SWATI) | V |
| 241 | HI 1418 (N.CHANDOUSI) | V |
| 242 | HI 1454 (ABHA) | V |
| 243 | HI 1479 | V |
| 244 | HI 1531 (HARSHITA) | V |
| 245 | HW 517 | ABL |
| 246 | HS 524 | ABL |
| 247 | HS 1097-17 (GIRIJA) | V |
| 248 | HS 1138-6-4 (SHAILJA) | V |
| 249 | HS 86 | V |
| 250 | HS 207 | V |
| 251 | HS 240 | V |
| 252 | HS 295 | V |
| 253 | HS 365 | V |
| 254 | HS 375 (HIMGIRI) | V |
| 255 | HD 2278 | V |
| 256 | HD 2281 | V |
| 257 | HD 2307 | V |
| 258 | HD 2385 | V |
| 259 | HD 2380 | V |
| 260 | HD 2402 | V |
| 261 | HD 2501 | V |
| 262 | HD 2610 | V |
| 263 | HD 2643 (GANGA) | V |
| 264 | HD 2687 (SHRESTH) | V |
| 265 | HD 2781 (ADITYA) | V |
| 266 | HD 2824 (POORVA) | V |
| 267 | HD 2833 | V |
| 268 | HD 2851 (PUSA VISHESH) | V |
| 269 | HD 2894 | V |
| 270 | HD 2987 | V |
| 271 | HD 3059 | V |
| 272 | HD 3090 | V |
| 273 | HD 4502 | ABL |
| 274 | HD 4530 | ABL |
| 275 | HDR 77 | ABL |
| 276 | HP 1209 | ABL |
| 277 | HD 1925 (SHERA) | V |
| 278 | HD 1949 (MOTI) | V |
| 279 | HD 1981 (PRATAP) | V |
| 280 | HD 2135 (NILGIRI) | V |
| 281 | HD 2177 | V |
| 282 | HD 2204 | V |
| 283 | HD 2236 | V |
| 284 | HD 2270 | V |
| 285 | HPW 42 (ARADHANA) | V |
| 286 | HPW 89 (SURABHI) | V |
| 287 | HPW 147 (PALAM) | V |
| 288 | HPW 155 | V |
| 289 | HPW 184 (CHANDRIKA) | V |
| 290 | HP 1102 | V |
| 291 | HP 1633 (SONALI) | V |
| 292 | HP 1731 (RAJLAKSHMI) | V |
| 293 | HP 1744 (RAJESWARI) | V |
| 294 | HP 1761 (JAGDISH) | V |
| 295 | HW 657 | V |
| 296 | HW 741 | V |
| 297 | HW 1085 (BHAWANI) | V |
| 298 | HW 1095 (COW-2) | V |
| 299 | HW 2004 (AMAR) | V |
| 300 | HW 2044 | V |
| 301 | HW 2045 | V |
| 302 | HW 5207 | V |
| 303 | HUW 12 (MALVIYA12) | V |
| 304 | HUW 37 (MALVIYA-37) | V |
| 305 | HUW 55 (MALVIYA55) | V |
| 306 | HUW 206 (MALVIYA206) | V |
| 307 | HUW 213 | V |
| 308 | HUW 318 | V |
| 309 | HUW 510 | V |
| 310 | HY 5 | LR |
| 311 | HYB 11 | LR |
| 312 | HY 12 | LR |
| 313 | HYB 65 | LR |
| 314 | HB 208 | LR |
| 315 | HYB 277 | LR |
| 316 | HYB 633 | LR |
| 317 | IWP 72 | LR |
| 318 | JI 7 | LR |
| 319 | JU 12 | LR |
| 320 | JWS 17 | LR |
| 321 | J 24 | LR |
| 322 | JAY | LR |
| 323 | JNK 4W-184 | LR |
| 324 | J 405 | LR |
| 325 | JOB 666 | LR |
| 326 | JW 3020 | LR |
| 327 | KRL 19 | V |
| 328 | KAILASH | V |
| 329 | KENPHAD 39 | V |
| 330 | K 68 | V |
| 331 | KALYANSONA | V |
| 332 | KSML 3 | V |
| 333 | K 0307 | V |
| 334 | K 0402 | V |
| 335 | K 0607 | V |
| 336 | K 7410 (SHEKHER) | V |
| 337 | K 9423 (UNNAT HALNA) | V |
| 338 | KENPHAD 25 | V |
| 339 | K 53 | V |
| 340 | K 88 (K8804) | V |
| 341 | K 816 | V |
| 342 | K 852 | V |
| 343 | K 7903 (HALNA) | V |
| 344 | K 8020 (TRIVENI) | V |
| 345 | K 8434 (PRASHAD) | V |
| 346 | K 8902 (INDRA) | V |
| 347 | K 9644 (ATAL) | V |
| 348 | K 9006 (UJIYAR) | V |
| 349 | K 9351 (MANDAKINI) | V |
| 350 | K 9465 (GOMTI) | V |
| 351 | LERMA RAJO | V |
| 352 | LAL BAHADUR | V |
| 353 | MACS 3949 | V |
| 354 | MP 1142 | V |
| 355 | MP 1201 | V |
| 356 | MP 1202 | V |
| 357 | MP 1203 | V |
| 358 | MP 3173 | V |
| 359 | MOTIA | V |
| 360 | MPO 1215 | V |
| 361 | MACS 6145 | V |
| 362 | MACS 6273 | V |
| 363 | MACS 6478 | V |
| 364 | MLKS 11 | V |
| 365 | MP 3288 | V |
| 366 | MP 4106 | V |
| 367 | MP 4010 | V |
| 368 | MPO 1106 | V |
| 369 | MACS 9 | V |
| 370 | MACS 2971 | V |
| 371 | MACS 3125 | V |
| 372 | MPO 215 | V |
| 373 | MP 3382 (JW3382) | V |
| 374 | NP 404 | LR |
| 375 | NP 832 | LR |
| 376 | NP 884 | LR |
| 377 | NP 12 | LR |
| 378 | NP 111 | LR |
| 379 | NP 852 | LR |
| 380 | NP 846 | LR |
| 381 | NP 809 | LR |
| 382 | NP 792 | LR |
| 383 | NP 125 | LR |
| 384 | NP 823 | LR |
| 385 | NP 775 | LR |
| 386 | NP 165 | LR |
| 387 | NP 824 | LR |
| 388 | NP 710 | LR |
| 389 | NP 52 | LR |
| 390 | NP 715 | LR |
| 391 | NP 737 | LR |
| 392 | NP 745 | LR |
| 393 | NP 761 | LR |
| 394 | NP 120 | LR |
| 395 | NP 718 | LR |
| 396 | NP 825 | LR |
| 397 | NP 818 | LR |
| 398 | NP 114 | LR |
| 399 | NP 770 | LR |
| 400 | NP 836 | LR |
| 401 | NP 890 | LR |
| 402 | NP 839 | LR |
| 403 | NP 101 | LR |
| 404 | NP 100 | LR |
| 405 | NI 5643 | V |
| 406 | NI 179 | V |
| 407 | NI 146 | V |
| 408 | NI 345 | V |
| 409 | NI 917 | V |
| 410 | NI 5749 | V |
| 411 | NI 747-19 | V |
| 412 | NIAW 917 (TAPOVAN) | V |
| 413 | NIAW 34 | V |
| 414 | NIAW 1994 (PHULE SAMADHAN) | V |
| 415 | NIAW 301 (TRIMBAK) | V |
| 416 | NIAW 1415 | V |
| 417 | NW 2036 | V |
| 418 | NW 5054 | V |
| 419 | NW 1014 | V |
| 420 | NW 1012 | V |
| 421 | NW 1067 | V |
| 422 | NARBADA 4 | V |
| 423 | NARMADA 112 | V |
| 424 | NARMADA 195 | V |
| 425 | NEPHAD 4 | V |
| 426 | N 59 | V |
| 427 | PBW 226 | V |
| 428 | PBW 590 | V |
| 429 | PBW 658 | V |
| 430 | PBW 660 | V |
| 431 | PBW 154 | V |
| 432 | PBW 120 | V |
| 433 | PBW 533 | V |
| 434 | PBW 138 | V |
| 435 | PBW 527 | V |
| 436 | PBW 34 | V |
| 437 | PBW 500 | V |
| 438 | PBW 596 | V |
| 439 | PBW 65 | V |
| 440 | PBW 222 | V |
| 441 | PBW 509 | V |
| 442 | PBN 51 | V |
| 443 | PBW 12 | V |
| 444 | PBW 396 | V |
| 445 | PBW 54 | V |
| 446 | PBW 443 | V |
| 447 | PBW 725 | V |
| 448 | PV 18 | V |
| 449 | PKV WASHIM (WSM1472) | V |
| 450 | TYPE II (PB TYPEII) | LR |
| 451 | UP 368 | V |
| 452 | UAS 304 | V |
| 453 | UTKALIKA | V |
| 454 | UP 1109 | V |
| 455 | UAS 446 | V |
| 456 | UAS 305 | V |
| 457 | UP 115 | V |
| 458 | UP 2003 | V |
| 459 | UP 2121 | V |
| 460 | UP 2526 | V |
| 461 | UP 215 | V |
| 462 | UP 2584 | V |
| 463 | UP 2572 | V |
| 464 | UP 2554 | V |
| 465 | UP 2565 | V |
| 466 | UP 2382 | V |
| 467 | UP 301 | V |
| 468 | RAJ 4037 | V |
| 469 | RSP 561 | V |
| 470 | RAJ MOL RODHAK | V |
| 471 | RAJ 821 | V |
| 472 | RAJ 6560 | V |
| 473 | RAJ 1972 | V |
| 474 | RAJ 3777 | V |
| 475 | RATAN (CG5016) | V |
| 476 | RIDLEY | Exotic lines |
| 477 | RW 3016 | V |
| 478 | RAJ 4079 | V |
| 479 | RAJ 1114 | V |
| 480 | RAJ 4083 | V |
| 481 | RW 346 | V |
| 482 | RAJ 4120 | V |
| 483 | RAJ 2184 | V |
| 484 | RAJ 6550 | V |
| 485 | SAGARIKA | V |
| 486 | SIDHI 2010 | V |
| 487 | SHARBATI SONORA | V |
| 488 | SKAML 1 | V |
| 489 | SKW 355 (Shalimar Wheat 2 ) | V |
| 490 | SKW 196 (SHALIMAR.W) | V |
| 491 | SONAK | V |
| 492 | SONORA 64 | V |
| 493 | SAFED LERMA | V |
| 494 | WH 331 | V |
| 495 | WH 157 | V |
| 496 | WH 283 | V |
| 497 | WH 416 | V |
| 498 | WH 912 | V |
| 499 | WH 896 | V |
| 500 | WH 291 | V |
| 501 | WH 711 | V |
| 502 | WH 533 | V |
| 503 | WH 1142 | V |
| 504 | WL 410 | V |
| 505 | WL 1562 | V |
| 506 | WL 2265 | V |
| 507 | WG 377 | V |
| 508 | WG 357 | V |
| 509 | WR 544 (PUSA GOLD) | V |
| 510 | WB 2 | V |
| 511 | VL 719 | V |
| 512 | VL 802 | V |
| 513 | VINATA (N 8223) | V |
| 514 | VL 404 | V |
| 515 | VL 804 | V |
| 516 | VL 832 | V |
| 517 | UAS 428 | V |
| 518 | BNI-1 | GS |
| 519 | BNI-2 | GS |
| 520 | BNI-3 | GS |
| 521 | BNI-4 | GS |
| 522 | BNI-5 | GS |
| 523 | BNI-6 | GS |
| 524 | Morocco | GS |
| 525 | LMPG | GS |
| 526 | CS2A/2M (Lr 28) | GS |
| 527 | SERI (MDCS 2496) | GS |
| 528 | Sr 24 (Tr 380-14) | GS |
| 529 | Sr 36 (Cook-2) | GS |
| 530 | Sr 36 (Cook) | GS |
| 531 | D 482 | GS |
| 532 | D 873 | GS |
| 533 | D 879 | GS |
| 534 | D 895 | GS |
| 535 | TL 2908 | GS |
| 536 | AMSEL CM 69191 | GS |
| 537 | CHIL/2*STAR/CMH112793-OTOPY-10M/2692-4 | GS |
| 538 | CHIL/2*STAR/CMH112793-OTOPY-10M/2692-2 | GS |
| 539 | MRNG/BUC"S"CLD"S"PLN"S"X12 | GS |
| 540 | ND/NG 9144//KAL/BB/3/CHIL | GS |
| 541 | SHANGHAI-3-260-104 | GS |
| 542 | SUZHOEF3ii1-23-04 | GS |
| 543 | TAB368.25//BUL//TURAACO | GS |
| 544 | VEE/3/237/GT12121/KL/BB/412 | GS |
| 545 | CMH2229-OTOY-1M/172/WH542CN079X21PRT | GS |
| 546 | WL6942 | GS |
| 547 | WL9241 | GS |
| 548 | CMH 751.66/2*CORM"S" | GS |
| 549 | MRNG BUC "S"PSN "S" | GS |
| 550 | WL 5442 | LR |
| 551 | W 809 | LR |
| 552 | W 1705 | LR |
| 553 | W 2052 | LR |
| 554 | W 3307 | LR |
| 555 | W 3339 | LR |
| 556 | W 5792 | LR |
| 557 | W 5793 | LR |
| 558 | W 6160 | LR |
| 559 | W 6191 | LR |
| 560 | W 6310 | LR |
| 561 | W 6370 | LR |
| 562 | W 6376 | LR |
| 563 | W 7340 | LR |
| 564 | W 7842 | LR |
| 565 | W 7993 | LR |
| 566 | W 8083 | LR |
| 567 | W 8436 | LR |
| 568 | W 8627 | LR |
| 569 | W 8699 | LR |
| 570 | H 567.71 | LR |
| 571 | PBW 343 | V |
| 572 | WL 6975 | LR |
| 573 | ALDAN | GS |
| 574 | CPAN 3045 | GS |
| 575 | HP 1531 | V |
| 576 | KBRL 10 | GS |
| 577 | KBRL 13 | GS |
| 578 | KBRL 22 | GS |
| 579 | HD 29 (new seed) | GS |
| 580 | HD 2 (new seed) | GS |
| 581 | W 485 | GS |
| 582 | CMH 77.308 | GS |
| 583 | NARADA-4 | GS |
| 584 | MONDHYA-32 | GS |
| 585 | NP-232 | GS |
| 586 | DBW-500 | GS |
| 587 | RAJ-4125 | GS |
| 588 | Lr-2c | GS |
| 589 | Lr-13sc | GS |
| 590 | Lr-23 | GS |
| 591 | Yr57/3*Gladius | GS |
| 592 | Sr26/2*Livington | GS |
| 593 | Sr26/2*Mace | GS |
| 594 | BAXI-288-18 | GS |
| 595 | NP-721 | GS |
| 596 | HYB-277 | GS |
| 597 | HYB-11 | GS |
| 598 | NP-799 | GS |
| 599 | RS-31-1 | GS |
| 600 | NP-830 | GS |
| 601 | GABD | GS |
| 602 | BAWA JI | GS |
| 603 | NP 4 | LR |
| 604 | TYPE 11 (PBC TYPE) | LR |
| 605 | VIJAY | GS |
| 606 | NP 771 | LR |
| 607 | K 65-missing CB | GS |
| 608 | BRNS 88-1 | MUTANTS |
| 609 | BRNS 88-2 | MUTANTS |
| 610 | BRNS 88-3 | MUTANTS |
| 611 | BRNS 88-4 | MUTANTS |
| 612 | BRNS 88-5 | MUTANTS |
| 613 | BRNS 88-6 | MUTANTS |
| 614 | BRNS 88-7 | MUTANTS |
| 615 | BRNS 88-8 | MUTANTS |
| 616 | BRNS 88-9 | MUTANTS |
| 617 | BRNS 88-10 | MUTANTS |
| 618 | BRNS 88-11 | MUTANTS |
| 619 | BRNS 88-12 | MUTANTS |
| 620 | BRNS 88-13 | MUTANTS |
| 621 | BRNS 88-14 | MUTANTS |
| 622 | BRNS 88-15 | MUTANTS |
| 623 | BRNS 88-16 | MUTANTS |
| 624 | BRNS 88-17 | MUTANTS |
| 625 | BRNS 88-18 | MUTANTS |
| 626 | BRNS 88-19 | MUTANTS |
| 627 | BRNS 88-20 | MUTANTS |
| 628 | BRNS 88-21 | MUTANTS |
| 629 | BRNS 88-22 | MUTANTS |
| 630 | BRNS 88-23 | MUTANTS |
| 631 | BRNS 88-24 | MUTANTS |
| 632 | KBRL10 | GS |
| 633 | KBRL13 | GS |
| 634 | HD 30 | GS |
| 635 | CMH77…. | GS |
| 636 | KBRL1 | GS |
| 637 | KBRL15 | GS |
| 638 | KBRL65 | GS |
| 639 | KBRL76 | GS |
| 640 | NAPHAL | LR |
| 641 | KHARCHIA LOCAL | LR |
| 642 | AC Domain | EXOTIC LINES |
| 643 | BHU35 | GS |
| 644 | BH1146 | GS |
| 645 | Chirya1 | GS |
| 646 | PBW 677 | V |
| 647 | PBW698 | GS |
| 648 | PBW701 | GS |
| 649 | PBW702 | GS |
| 650 | PBW703 | GS |
| 651 | PBW763 | GS |
| 652 | PBW765 | GS |

*V- Variety

ABL- Advance Breeding Line

GS- Genetic stock

LR- Landraces
